# Supplementary material for: MAIT cell activation is associated with disease severity markers in acute hantavirus infection
Source: Cell Rep Med. 2021 Mar 16;2(3):100220. doi: 10.1016/j.xcrm.2021.100220 (PMC7974553; doi:10.1016/j.xcrm.2021.100220)
Supplement: Document S1. Figures S1–S3 [file mmc1.pdf]

Cell Reports Medicine, Volume *2*

Supplemental information

MAIT cell activation is associated with disease severity markers in acute hantavirus infection

Kimia T. Maleki, Johanna Tauriainen, Marina García, Priscilla F. Kerkman, Wanda Christ, Joana Dias, Julia Wigren Byström, Edwin Leeansyah, Mattias N. Forsell, Hans-Gustaf Ljunggren, Clas Ahlm, Niklas K. Björkström, Johan K. Sandberg, and Jonas Klingström

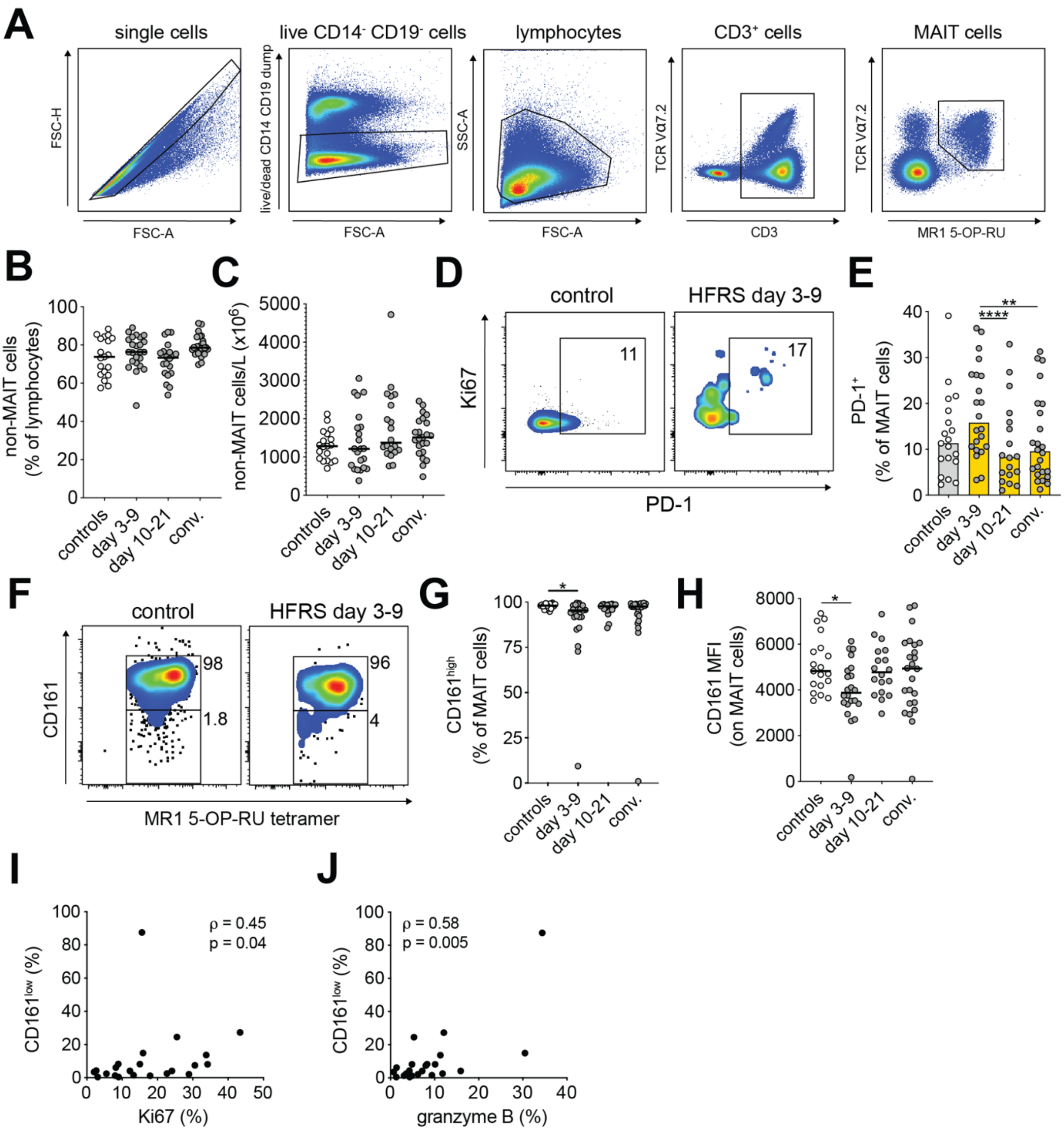

Figure S1. Gating strategy, non-MAIT T cell levels and MAIT cell surface marker expression in blood of HFRS patients and controls. Related to Figures 1-4. (A) Gating strategy for identification of MR1 5-OP-RU tetramer^{+} MAIT cells in PBMCs. (B) Frequency and (C) absolute count of non-MAIT T cells in controls and HFRS patients during acute, intermediate and convalescent phase. (D) Representative flow cytometry plots showing frequencies of PD-1^{+} MAIT cells in a control and in a patient with acute HFRS. (E) Frequencies of PD-1^{+} MAIT cells in controls and HFRS patients. (F) Representative flow cytometry plots of the frequencies of CD161^{high} and CD161^{low} MAIT cells in a control and a patient with acute HFRS. (G) Frequencies of CD161^{high} MAIT cells and (H) MFI of CD161 on MAIT cells in controls and HFRS patients. (I) Correlation between the frequency of CD161^{low} MAIT cells and the expression of Ki67 and (J) granzyme B on MAIT cells during acute HFRS. Acute phase, day 3-9 (n=22-24); intermediate phase, day 10-21 (n=18-21); convalescent phase (conv., n=24). Horizontal lines and bars represent median values. Kruskal Wallis test, Friedman test. $^{*}p < 0.05$, $^{**}p < 0.01$, $^{****}p < 0.0001$. Spearman's rank correlation coefficient.

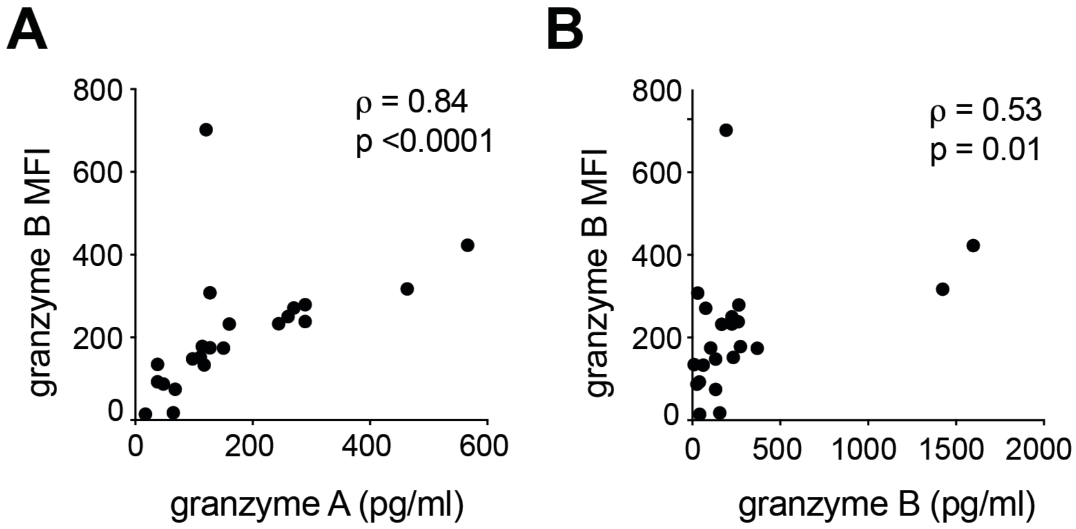

Figure S2. MAIT cell granzyme B expression correlates with levels of plasma granzymes. Related to Figures 2-3. n=22. Spearman's rank correlation coefficient.

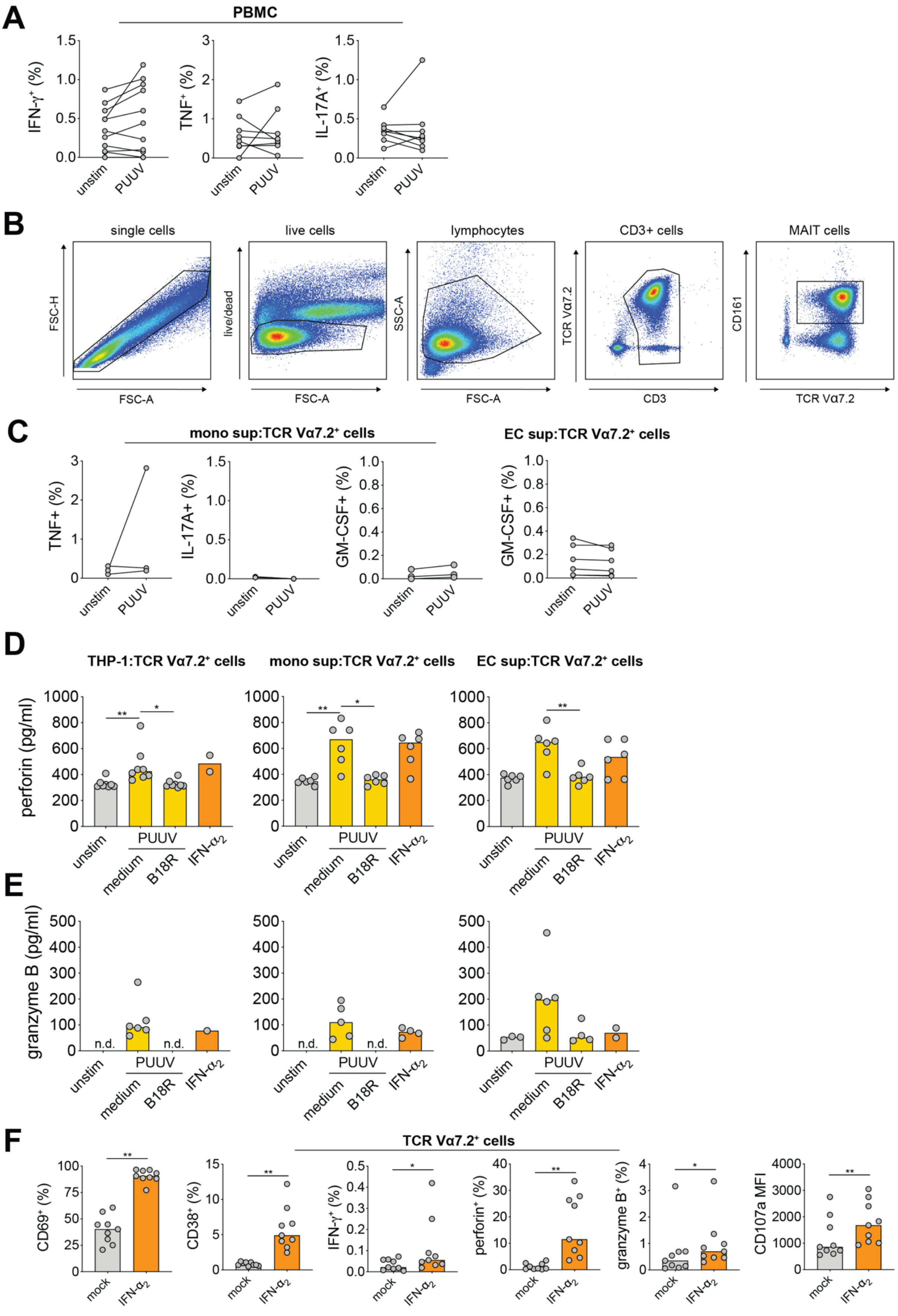

Figure S3. Gating strategy, cytokine expression and release of cytotoxic granule content by MAIT cells in *in vitro* studies. Related to Figures 5-6. (A) IFN-γ, TNF, and IL-17A expression on MAIT cells within PBMCs (n=8-11, at least three independent experiments) with or without PUUV for 72 h. (B) Gating strategy for

identification of MAIT cells among purified TCR Vα7.2$^+$ cells. (C) TNF, IL-17A, and/or GM-CSF expression on MAIT cells after culture with conditioned medium from primary monocytes (mono) (n=3, one experiment) or endothelial cells (EC) (n=6, two independent experiments). (D) Levels of perforin and (E) granzyme B released into supernatants from TCR Vα7.2$^+$ cells cultured with THP-1 cells (n=8, three independent experiments), supernatants from primary monocytes (n=6, two independent experiments) or endothelial cells (n=6, two independent experiments), or together with recombinant IFN-α_2 (1000 U/ml). Samples with undetectable granzyme B concentrations are not shown in the figure. (F) Expression of CD69, CD38, IFN-γ, perforin, granzyme B, and CD107a on MAIT cells (n=9, three independent experiments) with or without recombinant IFN-α_2 stimulation for 24 h. Bars represent median values. Wilcoxon test, Friedman test. *$p < 0.05$, **$p < 0.01$. n.d., not detected.
