# Supplementary material for: MAIT cell activation is associated with disease severity markers in acute hantavirus infection
Source: Cell Rep Med. 2021 Mar 16;2(3):100220. doi: 10.1016/j.xcrm.2021.100220 (PMC7974553; doi:10.1016/j.xcrm.2021.100220)
Supplement: Document S2. Article plus supplemental information [file mmc2.pdf]

Cell Reports Medicine

Article

MAIT cell activation is associated with disease severity markers in acute hantavirus infection

Graphical Abstract

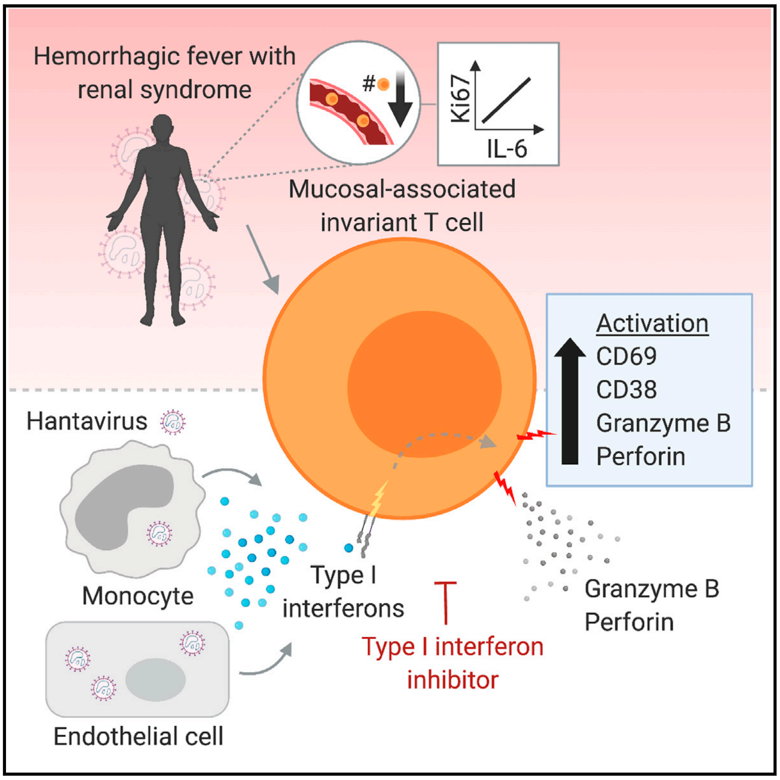

Highlights

- MAIT cells are activated in individuals with hemorrhagic fever with renal syndrome (HFRS)
- MAIT cell activation correlates with HFRS severity markers during hantavirus infection
- MAIT cell blood levels decline during acute HFRS
- Hantavirus-mediated MAIT cell activation is type I IFN dependent

Authors

Kimia T. Maleki, Johanna Tauriainen, Marina García, ..., Niklas K. Björkström, Johan K. Sandberg, Jonas Klingström

Correspondence

kimia.maleki@ki.se (K.T.M.),
jonas.klingstrom@ki.se (J.K.)

In brief

Maleki et al. demonstrate strong activation of mucosal-associated invariant T (MAIT) cells in individuals with hemorrhagic fever with renal syndrome (HFRS) caused by Puumala orthohantavirus. This phenotype is associated with markers of disease severity and is *in vitro* dependent on type I IFNs.

Maleki et al., 2021, Cell Reports Medicine 2, 100220
March 16, 2021 © 2021 The Author(s).
https://doi.org/10.1016/j.xcrm.2021.100220

CellPress

Cell Reports Medicine

CellPress
OPEN ACCESS

Article

MAIT cell activation is associated with disease severity markers in acute hantavirus infection

Kimia T. Maleki,[1,6,*] Johanna Tauriainen,[1] Marina García,[1] Priscilla F. Kerkman,[2,3] Wanda Christ,[1] Joana Dias,[1] Julia Wigren Byström,[2] Edwin Leeansyah,[1,4,5] Mattias N. Forsell,[2] Hans-Gustaf Ljunggren,[1] Clas Ahlm,[2] Niklas K. Björkström,[1] Johan K. Sandberg,[1] and Jonas Klingström[1,*]

[1]Center for Infectious Medicine, Department of Medicine Huddinge, Karolinska Institutet, Karolinska University Hospital, Stockholm, Sweden
[2]Department of Clinical Microbiology, Division of Infection & Immunology, Umeå University, Umeå, Sweden
[3]Department of Medical Microbiology, University Medical Center Utrecht, Utrecht, the Netherlands
[4]Tsinghua-Berkeley Shenzhen Institute, Tsinghua University, Shenzhen, China
[5]Programme in Emerging Infectious Diseases, Duke-National University of Singapore Medical School, Singapore, Singapore
[6]Lead contact
*Correspondence: kimia.maleki@ki.se (K.T.M.), jonas.klingstrom@ki.se (J.K.)
https://doi.org/10.1016/j.xcrm.2021.100220

SUMMARY

Hantaviruses are zoonotic RNA viruses that cause severe acute disease in humans. Infected individuals have strong inflammatory responses that likely cause immunopathology. Here, we studied the response of mucosal-associated invariant T (MAIT) cells in peripheral blood of individuals with hemorrhagic fever with renal syndrome (HFRS) caused by Puumala orthohantavirus, a hantavirus endemic in Europe. We show that MAIT cell levels decrease in the blood during HFRS and that residual MAIT cells are highly activated. This activation correlates with HFRS severity markers. *In vitro* activation of MAIT cells by hantavirus-exposed antigen-presenting cells is dependent on type I interferons (IFNs) and independent of interleukin-18 (IL-18). These findings highlight the role of type I IFNs in virus-driven MAIT cell activation and suggest a potential role of MAIT cells in the disease pathogenesis of viral infections.

INTRODUCTION

Hantaviruses are single-stranded negative-sense RNA viruses belonging to the Bunyavirales order. Humans are infected with hantaviruses upon inhalation of dust containing rodent excreta.[1] Depending on the hantavirus strain, infection can cause two different acute diseases: hemorrhagic fever with renal syndrome (HFRS) in Europe and Asia and hantavirus pulmonary syndrome (HPS) in the Americas. Despite high fatality rates of up to 16% and 40% for HFRS and HPS, respectively,[2,3] no specific treatment or US Food and Drug Administration (FDA)-approved vaccine is available. Puumala orthohantavirus (PUUV) is endemic in Europe and causes a relatively mild form of HFRS with a low fatality rate.[4,5] HFRS initially presents as flu-like symptoms, including fever, headache, and malaise. Many individuals also display kidney dysfunction and gastrointestinal symptoms such as abdominal pain, vomiting, diarrhea, and gastrointestinal bleeding.[2,6–11] The immune response to hantavirus infection is believed to play an important role in disease pathogenesis.[12] Hantavirus-infected individuals exhibit a strong inflammatory response with increased levels of pro-inflammatory cytokines[8,13–19] and strong cytotoxic responses with highly activated natural killer (NK) cells and CD8 T cells.[20–22] Moreover, endobronchial biopsies from individuals with HFRS have shown infiltration of CD8 T cells in lung and kidney tissue during acute disease.[23–25] However, the role of T cells in hantavirus pathogenesis is not fully understood, and hantavirus-specific CD8 T cell responses have been associated both with mild and severe disease.[26–28]

Mucosal-associated invariant T (MAIT) cells are innate-like T cells that are abundant in the blood and mucosal tissues such as the liver, gut, and lungs of healthy humans.[29] MAIT cells respond to microbial metabolite antigens derived from the riboflavin biosynthetic pathway of a range of bacteria and yeast species when presented by the major histocompatibility complex class I-related protein 1 (MR1) molecule.[30,31] MAIT cells can also be activated by cytokines independent of MR1.[32,33] Recently, reports have described activation of MAIT cells during acute viral infection caused by influenza virus, dengue virus, and severe acute respiratory syndrome coronavirus 2 (SARS-CoV-2) as well as during the acute stages of HIV-1 infection.[34–39] Furthermore, MAIT cell activation occurs in chronic viral infections caused by HIV-1, hepatitis B virus (HBV), hepatitis C virus (HCV), hepatitis D virus (HDV), and human T-lymphotropic virus 1.[37,40–46] Although interleukin-12 (IL-12) and IL-18 have been suggested as the main cytokines responsible for virus-mediated MAIT cell activation, IL-15 and type I interferons (IFNs) also play a role in this context.[33,34,47,48] Several studies have reported a decline in MAIT cell levels during acute viral infection,[34–36,38,39,47] suggesting dynamic regulation of MAIT cells during viral infection.

To better understand the human immune response to hantavirus infection and potential immunological events that can contribute to immunopathology, we characterized the peripheral

Check for updates

This is an open access article under the CC BY license (http://creativecommons.org/licenses/by/4.0/).

Table 1. Characteristics of HFRS patients and controls

	HFRS	Controls
Number of individuals	24	19
Gender (f/m)	9/15	6/13
Age (Y, mean ± SD)	49 ± 13	50 ± 9.4
WBC count ($\times 10^9$/L), median (range)[a]	7.4 (3.9–14.6)	nd
Platelet count ($\times 10^9$/L), median (range)[b]	73 (11–301)	nd
CRP (mg/L), median (range)[c]	45 (6–188)	nd
Serum creatinine (μmol/L), median (range)[d]	138 (55–960)	nd

HFRS, hemorrhagic fever with renal syndrome; WBC, white blood cell; nd, not done; CRP, C-reactive protein.
[a]WBCs; normal range, 3.5–8.8 × 10^9/L.
[b]Platelet count; normal range, 165–387 × 10^9/L for women and 145–348 × 10^9/L for men.
[c]CRP; reference value, less than 3 mg/L.
[d]Serum creatinine; reference value, less than 90 μmol/L for women and less than 105 μmol/L for men.

blood MAIT cell phenotype in 24 individuals with HFRS sampled longitudinally from early onset of symptoms until disease resolution. We show that MAIT cell levels are reduced in circulation during acute HFRS but recover during convalescence. Furthermore, the remaining MAIT cells display an activated phenotype with signs of ongoing proliferation, which is associated with HFRS disease severity markers. Finally, we show *in vitro* that PUUV-exposed primary monocytes and endothelial cells can activate MAIT cells and enhance their cytolytic potential in a type I IFN-dependent manner.

RESULTS

MAIT cell are reduced in acute HFRS

The MAIT cell compartment of PUUV-infected individuals with HFRS and matched uninfected controls (Table 1) was characterized in peripheral blood mononuclear cells (PBMCs) using flow cytometry. MAIT cells were dfined as $CD3^+$ MR1 5-(2-oxopropylideneamino)-6-D-ribitylaminouracil (5-OP-RU) $tetramer^+$ T cell receptor (TCR) $V\alpha7.2^+$ cells (see Figure S1A for the gating strategy). The median frequency of MAIT cells was reduced by 85% during acute HFRS (median, 0.26; interquartile range [IQR], 0.13%–0.69%; $p = 0.0012$) compared with controls (median, 1.7; IQR, 0.49%–2.54%) (Figures 1A and 1B). A similar reduction was also observed in absolute MAIT cell counts, with 96% fewer MAIT cells in acute HFRS compared with controls (Figure 1C). No such decrease occurred in non-MAIT T cells, defined by using a Boolean not-gate (Figures S1B and S1C), suggesting that there was no generalized lymphopenia. During the convalescent phase, MAIT cell frequencies were restored (Figure 1B), indicating that the reduction in circulating MAIT cells was transient.

Next, the relative frequencies of different MAIT cell subsets were studied. In healthy individuals, $CD8^+$ MAIT cells constitute the major MAIT cell subset, whereas a smaller fraction are CD4 and CD8 doublenegative (DN), and very few are $CD4^+$.[49,50] In individuals with HFRS, the $CD8^+$ MAIT cell subset was reduced compared with controls (Figures 1D and 1E). The decline in $CD8^+$ MAIT cells coincided with a relative increase in the DN MAIT cell subset on days 10–21 as well as in the minor $CD4^+$ MAIT cell subset on days 3–9 (Figures 1D and 1E). In addition, CD8 expression on $CD8^+$ MAIT cells was reduced on days 3–9 and 10–21 of HFRS compared with controls (Figure 1F). These findings show that PUUV infection causes a transient reduction in MAIT cell levels in the blood during the acute phase, with a concomitant change in subset distribution.

Residual MAIT cells in PUUV-infected individuals with HFRS are highly activated

Next, we assessed the activation status of residual MAIT cells still present in the circulation. MAIT cells of individuals with HFRS displayed an activated phenotype with increased expression of CD69, CD38, and granzyme B during days 3–9 of HFRS (Figures 2A and 2B). In addition, MAIT cells of affected individuals showed high expression of Ki67 during days 3–9, suggesting that the cells were proliferating (Figures 2A and 2B). On days 10–21 after onset of symptoms, expression of CD38, granzyme B, and Ki67 was still elevated, whereas CD69 expression had returned to similar levels as in controls (Figure 2B). Programmed cell death protein-1 (PD-1) expression in MAIT cells was increased during days 3–9 of HFRS compared with days 10–21 and convalescence (Figures S1D and S1E). Further, as described previously for activated MAIT cells in some settings,[41,51,52] CD161 expression was decreased in MAIT cells of individuals with HFRS on days 3–9 (Figures S1F–S1H). The increase in $CD161^{low}$ MAIT cells positively correlated with Ki67 and granzyme B expression in MAIT cells (Figures S1I and S1J). These data suggest that PUUV infection drives strong activation and proliferation of peripheral blood MAIT cells.

MAIT cell activation and proliferation are associated with disease severity markers

To investigate possible associations between MAIT cell activation during HFRS and aspects of the inflammatory response, plasma cytokine levels were measured. Compared with controls, individuals with HFRS displayed increased levels of IL-6, IL-10, IL-15, IL-18, tumor necrosis factor (TNF), IFN-γ, granzyme A, and granzyme B (Figures 3A and 3B). IL-1β, IL-12, IL-17A, IL-17C, IL-17E, and IL-22 were undetectable in all plasma samples. IL-2 was detected in a few individuals with HFRS and controls, but no difference was observed between the groups (data not shown). Although the levels of the MAIT cell-activating cytokines IL-15 and IL-18 were elevated during HFRS (Figure 3A), no correlation with MAIT cell activation markers was observed (data not shown). Remarkably, granzyme B median fluorescence intensity (MFI) in MAIT cells was positively correlated with granzyme A and B levels in plasma of individuals with HFRS (Figures S2A and S2B).

Low platelet counts and increased IL-6 levels have been associated with HFRS/HPS disease severity.[16,53,54] Interestingly, during acute HFRS, plasma IL-6 levels positively correlated with MAIT cell Ki67 expression (Figure 3C). In addition, IL-6 was positively correlated with granzyme B and PD-1 expression in MAIT cells (Figure 3C). Furthermore, expression of CD38 and Ki67 in MAIT cells of individuals with HFRS negatively correlated with the platelet count (Figure 3D). Collectively, this suggests

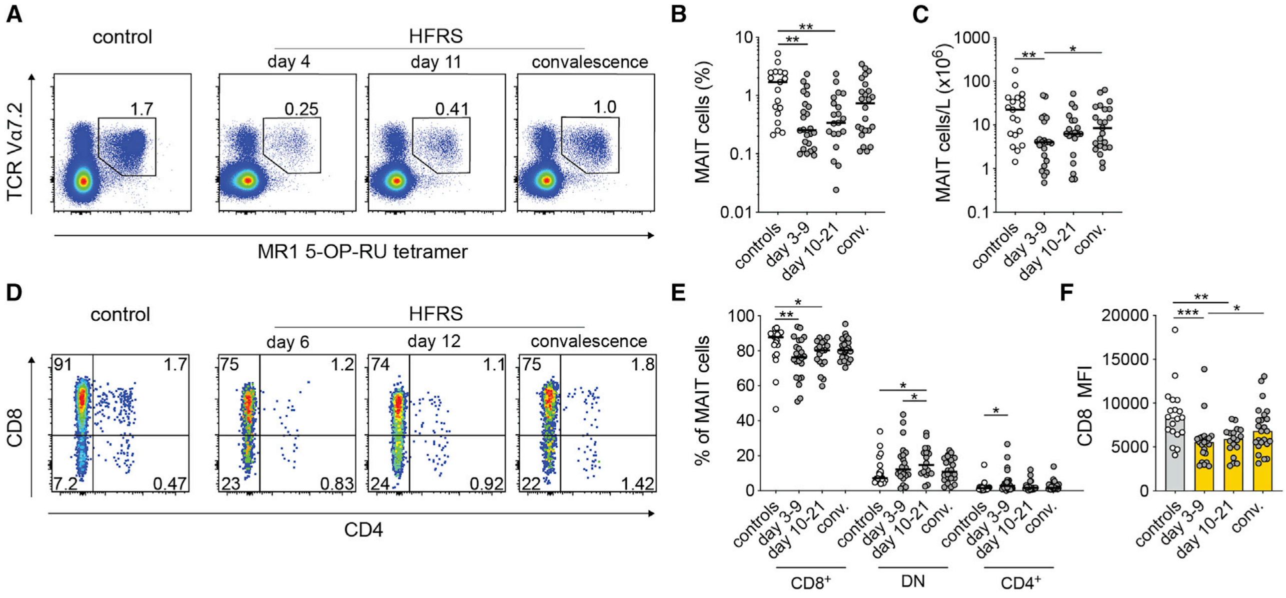

Figure 1. Blood MAIT cells are reduced during HFRS

(A) Representative flow cytometry plots showing frequencies of MAIT cells in a control and in an individual with HFRS (gated on $CD3^+$ cells).

(B) MAIT cell frequencies (gated on $CD3^+$ cells) in controls (n = 19) and individuals with HFRS during the acute phase (days 3–9, n = 24), intermediate phase (days 10–21, n = 21), and convalescent phase (conv.; n = 24).

(C) Absolute counts of MAIT cells in controls and individuals with HFRS during the acute phase (days 3–9, n = 22), intermediate phase (days 10–21, n = 20), and conv. (n = 23).

(D) Representative flow cytometry plots showing frequencies of MAIT cells expressing CD8 and CD4 a control and in an individual with HFRS.

(E) Frequencies of $CD8^+$, CD8 and CD4 double-negative (DN), and $CD4^+$ MAIT cells in controls and individuals with HFRS during the acute phase (days 3–9, n = 22), intermediate phase (days 10–21, n = 18), and conv. (n = 24).

(F) CD8 median fluorescence intensity (MFI) of $CD8^+$ MAIT cells of controls and individuals with HFRS during the acute phase (n = 22), intermediate phase (n = 18), and conv. (n = 24).

Horizontal lines and bars represent median values. Kruskal-Wallis test, Friedman test. *p < 0.05, **p < 0.01, ***p < 0.001.

that hantavirus-mediated MAIT cell activation may be linked to more severe disease.

MAIT cells of individuals with HFRS show an altered tissue homing profile

The transient decline in circulating MAIT cells observed during the acute phase of HFRS suggests that MAIT cells may redistribute from blood to tissue. We therefore assessed plasma levels of chemokines known to promote tissue migration as well as their cognate receptors on MAIT cells in circulation. Increased plasma levels of the CCR6 ligand CCL20 and the CCR9 ligand CCL25 during acute HFRS (Figure 4A) supported the notion that MAIT cells might have migrated to mucosal sites. To assess the homing profile of MAIT cells during HFRS, expression of CCR6, CCR9, and α4β7 integrin was evaluated by flow cytometry. Very low expression of CCR9 was detected in MAIT cells, and no significant differences in the levels of $CCR9^+$ MAIT cells were observed between the groups (Figure 4B). However, expression of α4β7 integrin on MAIT cells was reduced during days 3–9 and days 10–21 of HFRS (Figures 4C and 4D). Similarly, expression of CCR6 on MAIT cells was reduced during days 3–9 (Figures 4E and 4F). These data show increased levels of plasma chemokines associated with mucosal tissue homing concomitant with altered expression of homing markers on MAIT cells during HFRS. This could possibly reflect redistribution of MAIT cells from blood into tissues.

PUUV-exposed monocytic cells activate MAIT cells *in vitro*

Monocytes and dendritic cells are susceptible to PUUV infection *in vitro* and upregulate chemokine receptors and the co-stimulatory molecule CD86 after exposure to PUUV.[24] Myeloid cells exposed to other types of viruses have been shown previously to activate MAIT cells.[34] To investigate whether MAIT cells could be activated in a similar fashion by PUUV, PBMCs from healthy blood donors were exposed to PUUV at MOI 1 or left unstimulated. After 72 h, MAIT cell activation was evaluated by flow cytometry. MAIT cells in PUUV-exposed PBMCs were activated, as assessed by expression of CD69 (Figures 5A and 5B). However, MAIT cells did not upregulate IFN-γ, TNF, or IL-17A upon PUUV exposure (Figure S3A). To study in more detail the mechanisms of PUUV-mediated activation of MAIT cells, we established an *in vitro* system based on the human monocytic cell line THP-1 and purified TCR $V\alpha7.2^+$ cells from healthy blood donors. THP-1 cells were exposed to PUUV at MOI 5 or left unstimulated for 72 h, and TCR $V\alpha7.2^+$ cells were then added to the THP-1 cells. After 12 h of co-culture, supernatants were collected, and MAIT cells, here defined as TCR $V\alpha7.2^+$ $CD161^{high}$ $CD3^+$ cells (see Figure S3B for the gating strategy), were stained and assessed for activation by flow cytometry. MAIT cells co-incubated with PUUV-exposed THP-1 cells were clearly activated, as shown by increased expression of CD69, CD38, and CD25 at the level of frequency

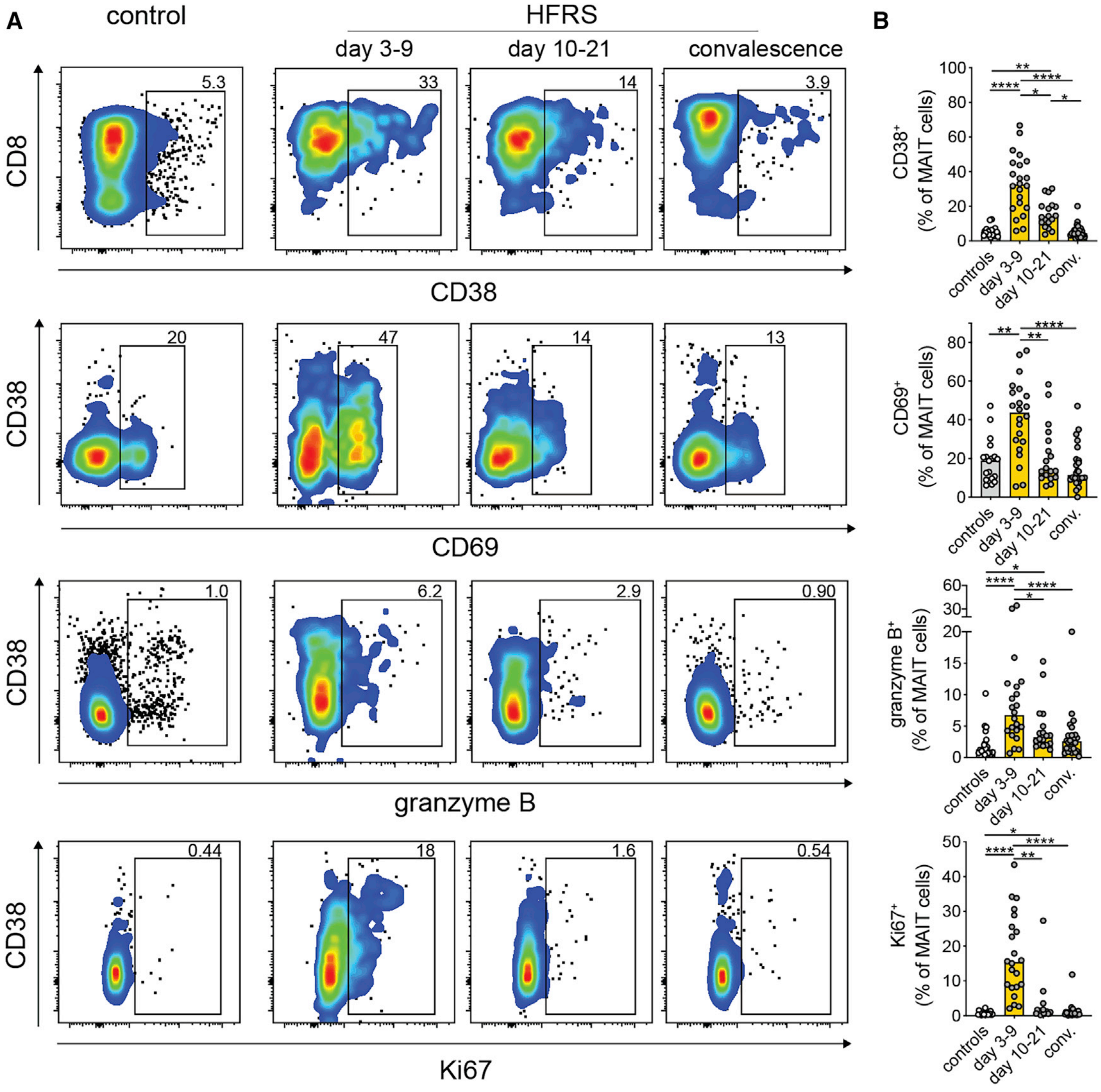

Figure 2. Residual MAIT cells are highly activated during HFRS

(A) Representative flow cytometry plots showing frequencies of CD38$^+$, CD69$^+$, granzyme B$^+$, and Ki67$^+$ MAIT cells in controls and individuals with HFRS during the acute phase (days 3–9, n = 22), intermediate phase (days 10–21, n = 18), and convalescent phase (conv.) (n = 24).

(B) Frequencies of CD38$^+$, CD69$^+$, granzyme B$^+$, and Ki67$^+$ MAIT cells in controls and individuals with HFRS during the acute phase (n = 22), intermediate phase (n = 18), and conv. (n = 24). Bars represent median values. Kruskal-Wallis test, Friedman test. *$p < 0.05$, **$p < 0.01$, ****$p < 0.0001$.

and/or MFI (Figures 5C–5F). Furthermore, MAIT cells incubated with PUUV-treated THP-1 cells also upregulated perforin, granzyme B, and CD107a, suggesting enhanced cytolytic capacity (Figures 5C–5F). However, no IFN-γ expression was detected in MAIT cells in response to PUUV-exposed THP-1 cells (Figures 5C–5F). These data indicate that monocytic cells exposed to PUUV can activate MAIT cells and increase their expression of cytolytic effector molecules.

PUUV-driven MAIT cell activation is dependent on soluble factors

To investigate whether MAIT cell activation was dependent on replicating virus, MAIT cells were co-incubated with THP-1 cells exposed to UV-inactivated PUUV. THP-1 cells exposed to UV-inactivated PUUV did not induce MAIT cell activation (Figure 6A), suggesting that THP-1-mediated MAIT cell activation is dependent on active PUUV replication. TCR-mediated activation of MAIT cells is dependent on recognition of MR1 presenting riboflavin metabolites, produced by many bacteria and certain yeast species.[31] To date, no virus-derived MR1-presented antigens have been described. Blocking of MR1 did not abrogate MAIT cell activation caused by PUUV-exposed THP-1 cells, indicating that PUUV-exposed THP-1 cells activate MAIT cells independent of MR1 (Figure 6B). Next we investigated whether the MAIT cell activation observed *in vitro* was dependent on cell-cell contact. To investigate this, conditioned medium (CM; i.e., supernatants from PUUV-exposed THP-1 cells) was collected and added to purified TCR Vα7.2$^+$ cells. After 12 h incubation,

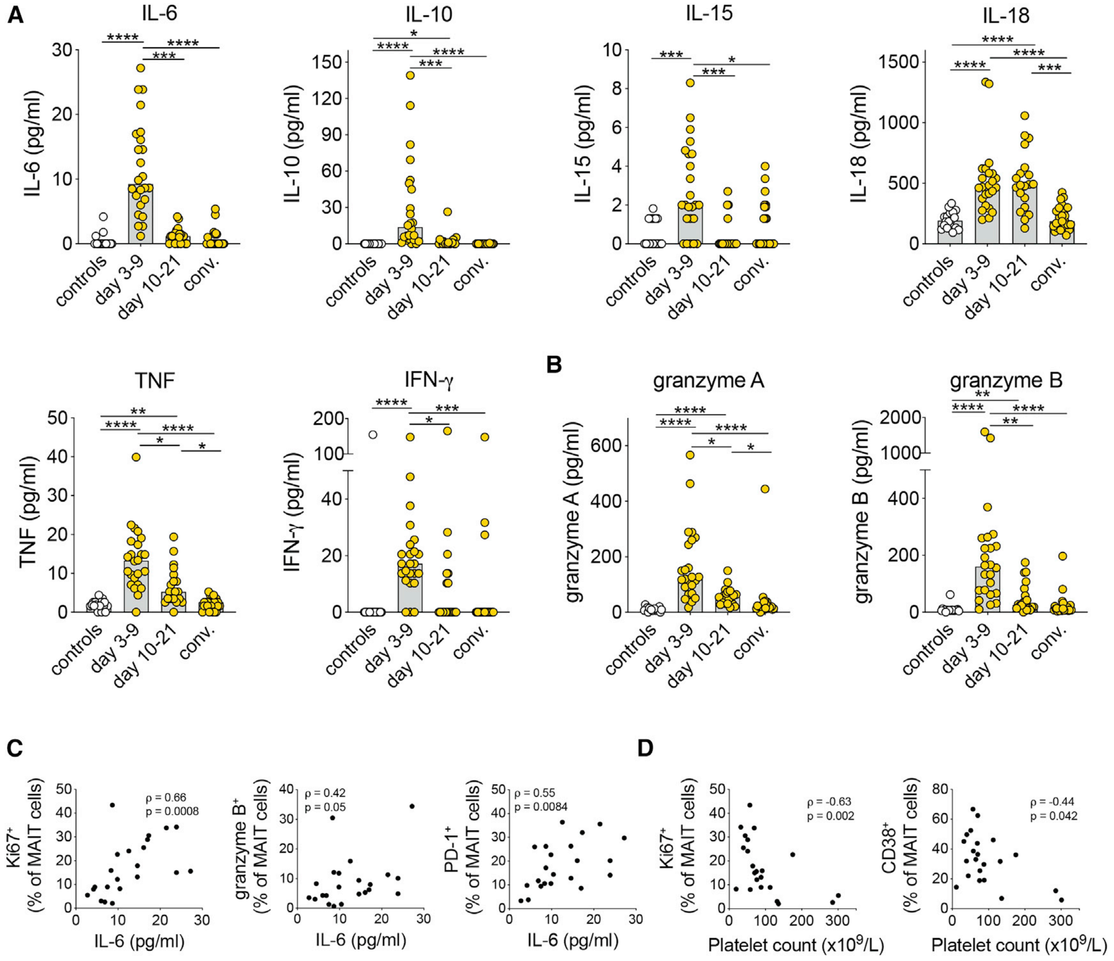

Figure 3. MAIT cell activation correlates with disease severity markers

(A and B) Plasma levels of (A) cytokines and (B) granzymes in controls (n = 18) and individuals with HFRS during the acute phase (days 3–9, n = 24), intermediate phase (days 10–21, n = 21), and convalescent phase (conv.) (n = 24).

(C) Correlations between plasma IL-6 levels and the frequencies of Ki67$^+$, granzyme B$^+$, and PD-1$^+$ MAIT cells during acute HFRS (n = 22).

(D) Correlations between platelet counts and the frequencies of Ki67$^+$ and CD38$^+$ MAIT cells during acute HFRS (n = 22).

Spearman's rank correlation coefficient. Bars represent median values. Kruskal-Wallis test, Friedman test. *p < 0.05, **p < 0.01, ***p < 0.001, ****p < 0.0001.

MAIT cell activation was evaluated. Interestingly, CM alone activated MAIT cells to levels similar to those seen after co-culture with THP-1 cells (Figure 6B), indicating that MAIT cell activation was dependent on soluble factors secreted by PUUV-exposed THP-1 cells. To identify which soluble factors were involved in activation of MAIT cells, we next analyzed cytokine levels in supernatants from PUUV-exposed and unstimulated THP-1 cells. Although no IL-6, IL-12, IL-15, or TNF could be detected in the supernatants, increased levels of IL-18 and IFN-α were detected upon PUUV exposure (Figures 6C and 6D).

PUUV-mediated MAIT cell activation is dependent on type I IFNs

Having shown that MAIT cell activation upon co-culture with PUUV-exposed THP-1 cells was dependent on soluble factors (Figure 6B), we proceeded with blocking experiments that targeted IL-18 and type I IFNs produced by PUUV-exposed THP-1 cells. Anti-IL-12 antibodies were also included in the assays to investigate any synergistic effects of IL-12 levels that may be below the detection limit of the ELISA. Although IL-18 levels were increased in THP-1 supernatants (Figure 6C), blocking IL-18 did not affect activation of MAIT cells co-cultured with PUUV-treated THP-1 cells (Figures 6E and 6F). In contrast, the type I IFN decoy receptor B18R efficiently blocked MAIT cell activation (Figures 6E and 6F), as assessed by inhibition of CD69 and perforin upregulation. In a few donors, a tendency toward a synergistic effect of IL-18 on perforin expression could be seen. However, overall, there was no significant reduction in MAIT cell activation after addition of anti-IL-18 or anti-IL-12 antibodies.

To confirm these findings in primary cells, monocytes enriched from healthy blood donors as well as human umbilical vein endothelial cells (HUVECs) were exposed to PUUV or left unstimulated. After 48 h, medium from these cells was collected and

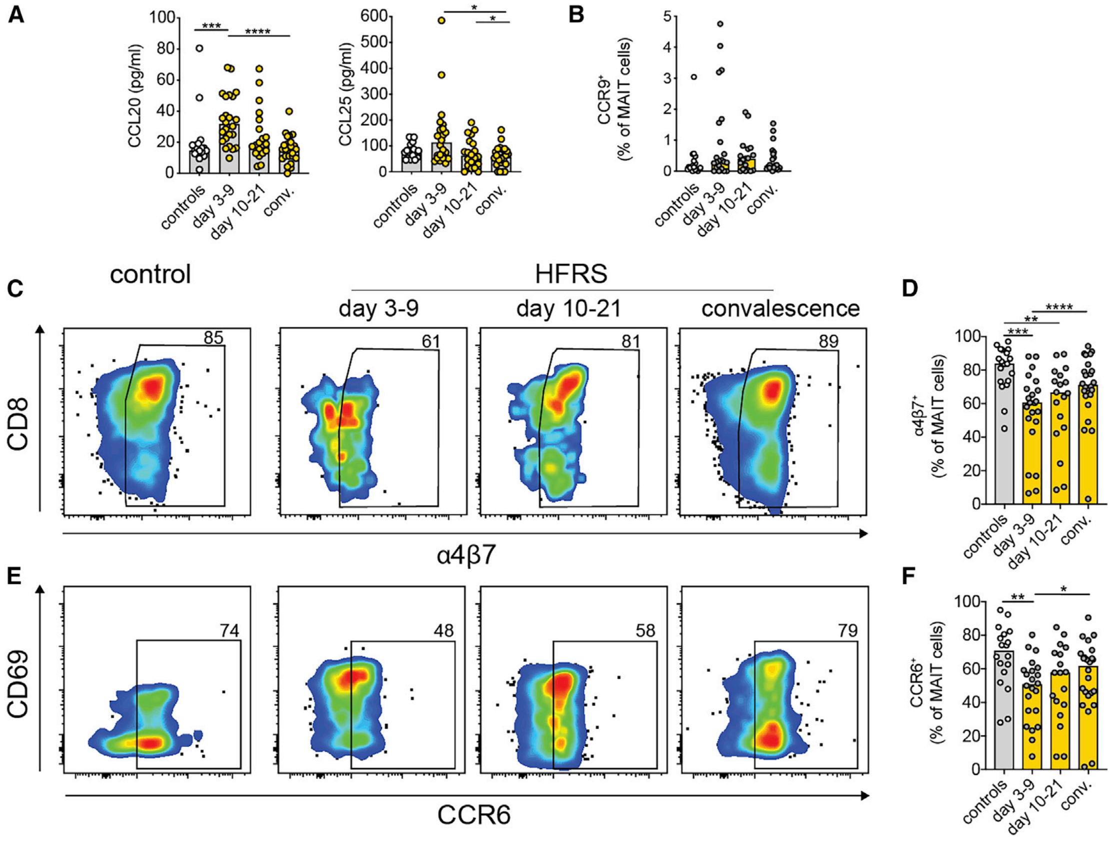

Figure 4. MAIT cells of individuals with HFRS display altered expression of tissue homing markers

(A) Plasma levels of CCL20 and CCL25 in controls (n = 18) and individuals with HFRS during the acute phase (days 3–9, n = 24), intermediate phase (days 10–21, n = 21), and convalescent phase (conv.) (n = 24).

(B) Frequencies of CCR9$^+$ MAIT cells in controls (n = 19) and individuals with HFRS during the acute phase (days 3–9, n = 22), intermediate phase (days 10–21, n = 18), and conv. (n = 24).

(C and D) Representative flow cytometry plots (C) and frequencies (D) of α4β7$^+$ MAIT cells in controls and individuals with HFRS.

(E and F) Representative flow cytometry plots (E) and frequencies (F) of CCR6$^+$ MAIT cells in controls and individuals with HFRS during the acute phase (n = 22), intermediate phase (n = 18), and conv. (n = 24).

Bars represent median values. Kruskal-Wallis test, Friedman test. *p < 0.05, **p < 0.01, ***p < 0.001, ****p < 0.0001.

transferred to purified TCR Vα7.2$^+$ cells. After 24 h culture, MAIT cell activation was assessed by flow cytometry. Supernatants from PUUV-exposed primary monocytes and HUVECs activated MAIT cells in a similar fashion as THP-1 cells (Figures 6G–6J). This activation was abrogated by B18R (Figures 6G–6J). As observed for THP-1 cells, primary cells exposed to PUUV did not significantly increase IFN-γ expression in MAIT cells (Figures 6G and 6H). However, a tendency toward slightly increased expression of IFN-γ could be seen after incubation with supernatants from PUUV-exposed monocytes (Figures 6G and 6H). In general, however, very little cytokine expression was detected in MAIT cells after culture with supernatants from monocytes or HUVECs (Figure S3C).

The increased expression of perforin, granzyme B, and CD107a in MAIT cells activated by PUUV-exposed cells prompted us to analyze the levels of perforin and granzyme B in the culture supernatants following stimulation with THP-1 cells, primary monocytes, or HUVECs. Interestingly, type I IFN-dependent release of perforin and granzyme B was detected in all three model systems, confirming that MAIT cells degranulate (Figures S3D and S3E). In support of previous findings by Lamichhane et al.,[48] we confirmed that type I IFNs alone can activate MAIT cells (Figure S3F). Collectively, these data show that PUUV-exposed cells activate MAIT cells in a type I IFN-dependent manner, largely independent of IL-18.

Finally, to explore the physiological relevance of IFN-mediated MAIT cell activation during PUUV infection, plasma levels of IFN-α were analyzed in individuals with HFRS. Interestingly, and in contrast to previous reports,[20,55] increased IFN-α levels were observed during acute HFRS compared with convalescence and controls (Figure 6K). These findings suggest a role of type I IFNs in MAIT cell activation during PUUV infection.

DISCUSSION

In recent years, multiple studies have reported MAIT cell activation during viral infection.[34,35,38–42,46,47,56] Here we characterized MAIT cell responses in the context of human hantavirus

CellPress
OPEN ACCESS

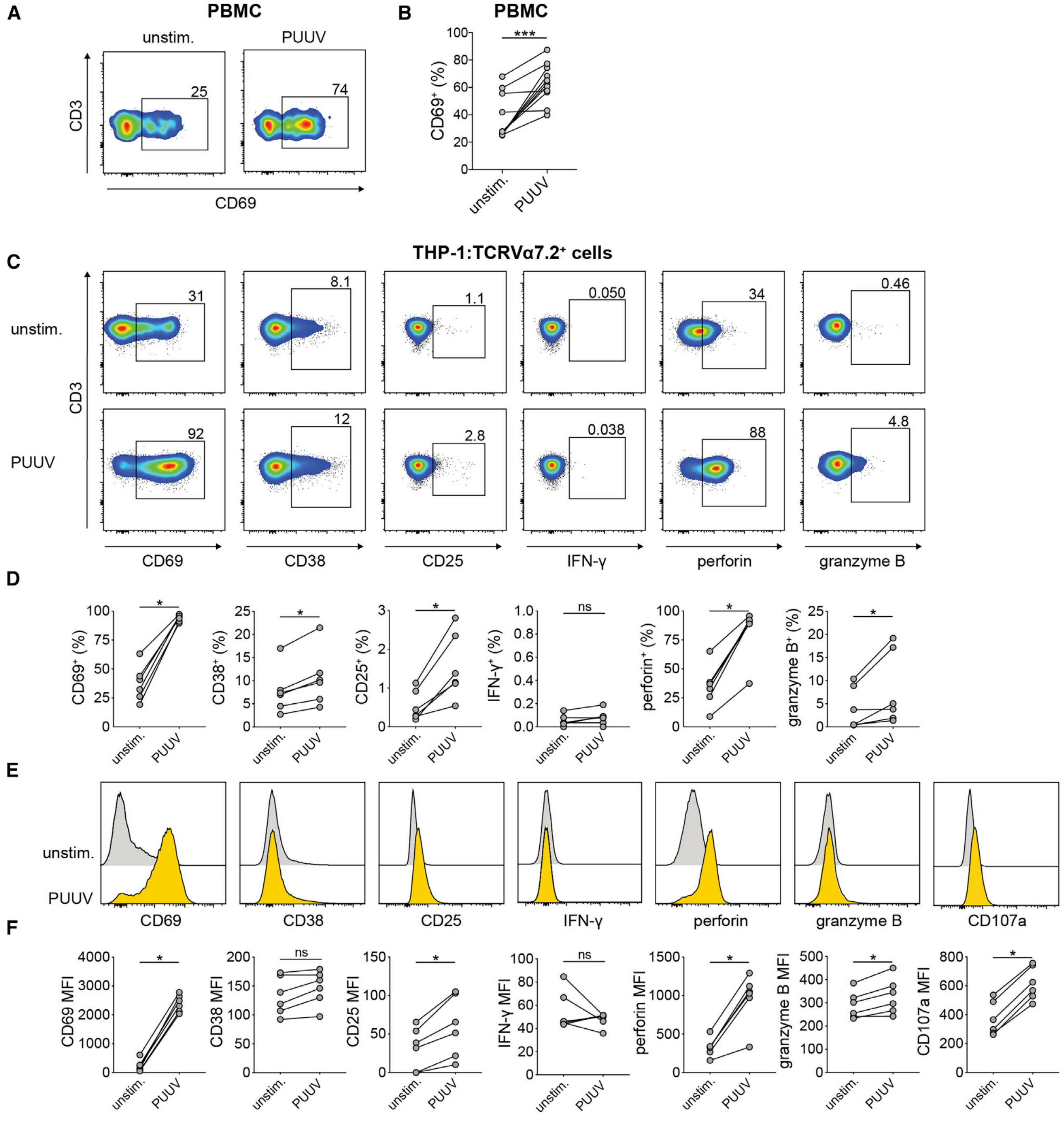

Figure 5. PUUV-exposed antigen-presenting cells stimulate MAIT cell activation *in vitro*

(A and B) PBMCs from blood donors were exposed to PUUV (MOI 1) or left unstimulated for 72 h, and then MAIT cells were assessed for CD69 expression (n = 11, four independent experiments).

(C–F) THP-1 cells were treated with PUUV (MOI 5) or left unstimulated for 72 h and then co-incubated with TCR Vα7.2$^+$ cells purified from buffy coats from blood donors for 12 h.

(C and D) Representative flow cytometry plots (C) and graphs (D) showing the frequencies of CD69$^+$, CD38$^+$, CD25$^+$, IFN-γ^+, perforin$^+$, and granzyme B$^+$ MAIT cells after 12 h co-incubation with unstimulated and PUUV-exposed THP-1 cells (n = 6, two independent experiments).

(E and F) Representative histograms (E) and graphs (F) displaying the mean fluorescence intensity (MFI) of CD69, CD38, CD25, IFN-γ, perforin, granzyme B, and CD107a in MAIT cells after 12 h co-incubation with unstimulated and PUUV-exposed THP-1 cells (n = 6, same donors as in C and D, two independent experiments).

Bars represent median values. Wilcoxon signed-rank test. $^*p < 0.05$, $^{***}p < 0.001$.

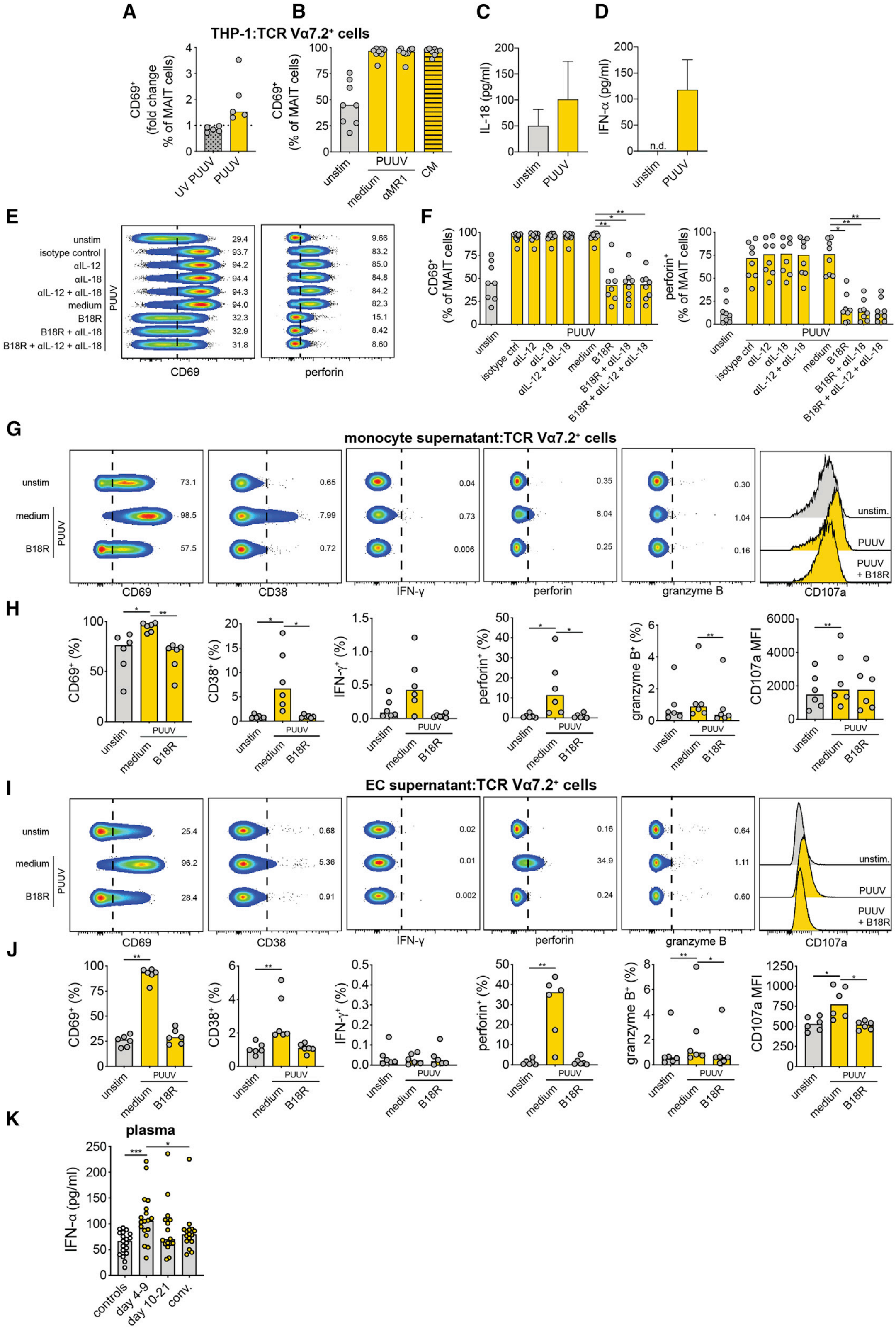

(legend on next page)

infection. We found that MAIT cell levels were reduced in blood during acute hantavirus-induced HFRS and that residual MAIT cells were highly activated and displayed an altered homing phenotype. Further, we dissected the mechanisms behind MAIT cell activation *in vitro* and showed that PUUV-exposed monocytes and endothelial cells can activate MAIT cells in a type I IFN-dependent manner.

Hantaviruses cause severe systemic infection in humans, characterized by high levels of inflammatory cytokines associated with disease severity.[16,53] Affected individuals also show strong activation and proliferation of various immune cells.[20,21,57] This, together with the observation that hantaviruses are not cytopathic,[58,59] suggests that HFRS and HPS are, to a significant degree, immune-mediated diseases.[12] $CD8^+$ T cells and NK cells have been shown previously to be activated in humans infected with hantavirus.[20,21,28] Here we found that MAIT cells of individuals with HFRS are highly activated, as assessed by expression of CD38, CD69, and granzyme B. MAIT cell activation has been reported previously with several other acute viral infections.[34–37] Here we also show that PUUV infection leads to MAIT cell proliferation, as defined by Ki67 expression. Remarkably, in a third of the patients, 20% or more of the MAIT cells were $Ki67^+$. Although increased Ki67 expression in MAIT cells has been reported recently in severe coronavirus disease 2019 (COVID-19),[35] the magnitude of this response seems to be greater in individuals with HFRS. The present findings add to previous reports of MAIT cell responses in viral infection and suggest that MAIT cell activation is an integral part of the immune response upon viral infection.

Whether MAIT cells are bystanders or active participants in HFRS pathogenesis remains to be investigated. In dengue virus infection, expression of CD38 on MAIT cells was higher in individuals who progressed to dengue hemorrhagic fever, suggesting that MAIT cell activation is associated with a more severe disease.[34] Recently, we reported an association between CD69 expression on MAIT cells and COVID-19 outcome.[35] In COVID-19, Ki67 expression is also higher in severe compared with moderate disease.[35] In another study, however, CD69 expression of MAIT cells was reported to be higher in individuals with COVID-19 with early as opposed to late hospital discharge, highlighting the possibility that, in some instances, MAIT cells may have a protective role in individuals with milder disease.[36] In mice, MAIT cells seem to have a protective role during influenza virus infection by promoting T cell enrichment at infected sites and enhancing cytokine production.[44,47] The correlations found here between plasma IL-6 levels and expression of Ki67, granzyme B, and PD-1 on MAIT cells of individuals with HFRS may not necessarily indicate a causal association but suggest that individuals with strong inflammatory responses also exhibit potent MAIT cell responses. In line with this, MAIT cell activation has been associated previously with plasma levels of IL-6 in individuals with systemic lupus erythematosus and COVID-19.[38,60] We also detected a negative correlation between platelet count and frequencies of $Ki67^+$ and $CD38^+$ MAIT cells. Elevated peripheral IL-6 levels and decreased platelet counts have been associated with increased disease severity in HFRS and HPS.[16,53,54] These data suggest that individuals with more severe disease exhibit stronger MAIT cell activation. This could reflect a stronger inflammatory milieu that drives MAIT cell activation. It is also possible that MAIT cells contribute to HFRS pathogenesis by producing pro-inflammatory cytokines. Such a scenario would be consistent with the recently described role of MAIT cells as early drivers of the cytokine response during streptococcal toxic shock syndrome.[61] Whether MAIT cells contribute to disease resolution or progression during HFRS remains to be elucidated.

Activated MAIT cells can produce pro-inflammatory cytokines such as IFN-γ, TNF, IL-17A, and GM-CSF.[29] Cytokine-mediated MAIT cell activation primarily induces IFN-γ production in MAIT cells.[32] This has also been seen upon virus-mediated MAIT cell activation.[44,47] In the *in vitro* assays performed here, PUUV-activated MAIT cells did not show a significant increase in IFN-γ or any other cytokine. This is in line with previous reports showing that type I IFNs alone fail to induce cytokine expression in MAIT cells.[34,48] The levels of IL-18 detected in the supernatants of PUUV-exposed THP-1 cells appeared to be insufficient for induction of MAIT cell IFN-γ production. However, it is possible that the

Figure 6. PUUV-induced MAIT cell activation is dependent on type I IFNs

(A) CD69 expression (fold change normalized to unstimulated, depicted by a dotted line) on MAIT cells after co-incubation with THP-1 cells exposed to PUUV or UV-inactivated PUUV (n = 5, two independent experiments).

(B) CD69 expression on MAIT cells after co-incubation with THP-1 cells exposed to PUUV in the presence or absence of anti-MR1 and MAIT cells incubated with conditioned medium (CM) from PUUV-exposed THP-1 cells (n = 8, three independent experiments).

(C and D) Levels of IL-18 (C) and IFN-α (D) in supernatants of unstimulated and PUUV-exposed THP-1 cells 72 h after exposure (median + SD of three independent experiments).

(E and F) Anti-IL-12 antibody and/or anti-IL-18 antibody and/or the type I IFN blocking reagent B18R was added to THP-1 cultures prior to addition of TCR Vα7.2$^+$ cells (n = 8, same donors as in B, three independent experiments).

(E) Representative flow cytometry plots of CD69 and perforin expression on MAIT cells following 12 h co-incubation with PUUV-exposed or unstimulated THP-1 cells.

(F) Frequencies of $CD69^+$ and (H) $perforin^+$ MAIT cells.

(G–J) Primary monocytes (n = 2) or endothelial cells (ECs) were treated with PUUV (MOI 1) or left unstimulated for 48 h and then co-incubated with purified TCR Vα7.2$^+$ cells for 24 h.

(G and H) Representative flow cytometry plots (G) and graphs (H) showing the expression of CD69, CD38, IFN-γ, granzyme B, perforin, and CD107a on MAIT cells (n = 6, two independent experiments) following 24 h incubation with CM from PUUV-exposed or unstimulated primary monocytes with or without B18R.

(I and J) Representative flow cytometry plots (I) and graphs (J) showing the expression of CD69, CD38, IFN-γ, perforin, granzyme B, and CD107a on MAIT cells (n = 6, two independent experiments) following 24 h incubation with CM from PUUV-exposed or unstimulated primary ECs with or without B18R.

(K) IFN-α levels in plasma of controls (n = 19) and individuals with HFRS during the acute phase (days 3–9, n = 18), intermediate phase (days 10–21, n = 16), and conv. n = 16).

Bars represent median values. Kruskal-Wallis test, Friedman test; median. n.d., not detected. *$p < 0.05$, **$p < 0.01$, ***$p < 0.001$.

increased IL-18 levels, or other soluble factors, in individuals with HFRS may stimulate MAIT cells to produce IFN-γ *in vivo*. Hence, activated MAIT cells in individuals with HFRS could potentially contribute to the pro-inflammatory cytokine response.

Although PUUV-mediated MAIT cell activation *in vitro* did not induce a clear cytokine response, it did cause upregulation and release of perforin and granzyme B. These data are corroborated by recent findings showing that cytokine-activated MAIT cells can degranulate[32,48] and suggests that virus-primed MAIT cells show increased cytotoxic capacity. In individuals with HFRS, increased granzyme B expression in MAIT cells was observed during the acute phase. Intriguingly, granzyme B expression in MAIT cells was positively correlated with plasma levels of granzyme A and B. This suggests a possible link between MAIT cell degranulation and increased granzyme levels in the blood. Although the function of extracellular granzymes is not fully understood, a growing body of evidence points toward them having a pro-inflammatory role.[62–64] Moreover, granzyme B has been reported to cleave extracellular proteins important for cell-cell integrity.[65,66] This may suggest a possible role of extracellular granzyme B in causing increased vascular permeability,[65,66] a common hallmark of hantavirus infection.[5]

The finding that PUUV-mediated MAIT cell activation *in vitro* is dependent on type I IFNs suggests a mechanism distinct from what has been proposed for HCV, dengue virus, and influenza virus, where IL-18 has been suggested as the key cytokine driving MAIT cell activation.[34,47] A synergistic role of type I IFNs has been shown in HCV- and influenza virus-mediated MAIT cell activation.[34,47,48] Type I IFNs have also been shown to have a co-stimulatory function, boosting MAIT cell responses upon MR1-dependent activation.[48] Here we show that PUUV-driven MAIT cell activation is dependent on type I IFNs. However, it should be noted that, although MAIT cell activation *in vitro* was dependent on type I IFNs, it is possible that other cytokines contribute to activation in individuals with HFRS.

We observed significant decline in blood MAIT cells during acute HFRS. No reduction was seen in non-MAIT T cells, indicating a degree of cell type specificity in this decline. Nevertheless, it is possible that other innate-like T cell subsets are also affected in a similar manner as MAIT cells. Loss of blood MAIT cells has been observed previously in infection with HIV, HCV, HBV-HDV, influenza virus, HTLV-1, and SARS-CoV-2,[34–36,38–40,43,45,56] suggesting that this is common in many viral infections. Similar to what has been seen for influenza virus and SARS-CoV-2 infection,[35,39,47] the reduction of MAIT cells in the blood of individuals with HFRS appeared to be transient. This is in contrast to what has been seen in chronic viral infection with HIV and HCV, where MAIT cell loss seems to be permanent.[40,41,43,56] The observation that blood MAIT cells levels of individuals with HFRS were restored at convalescence indicates that MAIT cells are not depleted. A possible explanation for the transient decline in blood MAIT cells is redistribution to specific tissues, as reported recently for monocytes and dendritic cells during HFRS.[24] Here we observed increased plasma levels of CCL20 during acute HFRS, which could potentially drive re-distribution of CCR6$^+$ MAIT cells from the blood to mucosal sites. Indeed, residual blood MAIT cells of individuals with HFRS showed an altered homing profile where the frequencies of cells expressing the mucosal tissue homing marker CCR6 and the gut homing marker $\alpha4\beta7$ integrin were reduced.

Additional analyses of the residual MAIT cells showed that the CD8$^+$ MAIT cell compartment was reduced in frequency during acute HFRS. Similar findings have been reported for HBV-HDV-co-infected individuals and individuals with COVID-19.[38,42] The observed downregulation of CD8 on CD8$^+$ MAIT cells of HFRS could be one explanation for this reduction and may also explain the relative increase in the DN MAIT cell compartment.[50] Moreover, because CD8$^+$ MAIT cells seem to express higher levels of $\alpha4\beta7$ integrin than DN MAIT cells,[50] it is possible that CD8$^+$ MAIT cells are more prone to migrate to tissue. Further, given that CD8$^+$ MAIT cells produce IFN-γ and TNF to a higher degree compared with DN and CD4$^+$ MAIT cells,[50,67] one can speculate that CD8$^+$ MAIT cells might contribute to local inflammation at sites where they may be enriched during HFRS.

In conclusion, we show that MAIT cells are reduced in the blood during HFRS and that remaining MAIT cells are highly activated. The observed correlations between MAIT cell activation and markers of disease severity suggest that more severe disease may be associated with higher MAIT cell activation. Further, we show that PUUV-mediated activation of MAIT cells *in vitro* is dependent on type I IFNs, independent of IL-18, and does not trigger MAIT cell cytokine production. PUUV-mediated MAIT cell activation does, however, increase the cytolytic potential of MAIT cells, which may potentially promote inflammation.

Limitations of study

One limitation of this study is the relatively small study population. Although HFRS caused by PUUV often leads to hospitalization, the disease course is rarely very severe. A larger study population might have allowed stratification of individuals into different disease severity groups and revealed direct associations between MAIT cell activation markers and disease severity. Such analyses are likely easier to address in studies including individuals with HPS. Investigation of MAIT cell responses in individuals with HPS would also allow analyses of associations with fatality. The analyses in this study were limited by sample availability. Given the low MAIT cell levels in individuals with HFRS, a larger sample volume (i.e., more PBMCs from patients) would have allowed functional studies aiming to describe the cytokine production capacity of MAIT cells during HFRS. Furthermore, access to tissue samples, such as biopsies from the lungs or gut, would permit assessment of MAIT cell infiltration into tissues. We showed that PUUV-driven MAIT cell activation was dependent on type I IFNs in three different *in vitro* infection models. However, none of these models fully mimics the cytokine milieu in peripheral blood of individuals with HFRS. Further studies using 3D infection models or serum-based models may add to the understanding of how hantaviruses modulate MAIT cell responses.

STAR★METHODS

Detailed methods are provided in the online version of this paper and include the following:

- KEY RESOURCES TABLE

- RESOURCE AVAILABILITY
  - Lead contact
  - Materials availability
  - Data and code availability
- EXPERIMENTAL MODEL AND SUBJECT DETAILS
  - Patient samples
  - Cells and viruses
- METHOD DETAILS
  - *In vitro* PUUV stimulation of PBMCs
  - *In vitro* stimulation of MAIT cells using THP-1 cells
  - *In vitro* stimulation of MAIT cells using primary cell supernatants
  - Biotinylation assay
  - Flow cytometric analyses
  - Multiplex immunoassay
  - ELISA
- QUANTIFICATION AND STATISTICAL ANALYSIS

SUPPLEMENTAL INFORMATION

Supplemental Information can be found online at https://doi.org/10.1016/j.xcrm.2021.100220.

ACKNOWLEDGMENTS

This study was supported by grants from the Swedish Foundation for Strategic Research (project SB12-0003), the Swedish Research Council (projects K2015-56X-22774-01-3 and 2018-02646), the County Council of Västerbotten, Umeå University, and Karolinska Institutet. J.D. was supported by Fundação para a Ciência e a Tecnologia doctoral fellowship SFRH/BD/85290/2012, co-funded by the Programa Operacional Potencial Humano-Quadro de Referência Estratégico Nacional and the European Social Fund. The MR1 tetramer technology was developed jointly by Dr. James McCluskey, Dr. Jamie Rossjohn, and Dr. David Fairlie, and the material was produced by the NIH Tetramer Core Facility and permitted to be distributed by the University of Melbourne (task order 34348).[30] The α4β7 antibody was obtained through the NIH AIDS Reagent Program, Division of AIDS, NIAID, NIH: Anti-human α4-β7 integrin (Act-1) (catalog number 11718) from Dr. A.A. Ansari.[68] PUUV strain CG-1820 was kindly provided by Prof. Richard M. Elliott (University of Glasgow). A549-V cells were kindly provided by Richard Randall (University of St Andrews). The graphical abstract was created with BioRender.

AUTHOR CONTRIBUTIONS

Conceptualization, K.T.M., N.K.B., J.K.S., and J.K.; validation, K.T.M.; formal analysis, K.T.M.; investigation, K.T.M., J.T., M.G., P.F.K., and W.C.; resources, C.A. and J.W.B.; methodology, K.T.M., J.T., J.D., E.L., N.K.B., and J.K.; visualization, K.T.M.; writing – original draft, K.T.M. and J.K.; writing – review & editing, K.T.M., J.T., M.G., P.F.K., W.C., J.D., J.W.B., E.L., M.N.F., H.-G.L., C.A., N.K.B., J.K.S., and J.K.; project administration, M.N.F., H.-G.L., C.A., and J.K.; supervision, M.N.F., H.-G.L., C.A., and J.K.; funding acquisition, C.A., H.-G.L., and J.K.

DECLARATION OF INTERESTS

The authors declare no competing interests.

Received: April 20, 2020
Revised: December 21, 2020
Accepted: February 19, 2021
Published: March 16, 2021

REFERENCES

1. Vaheri, A., Henttonen, H., Voutilainen, L., Mustonen, J., Sironen, T., and Vapalahti, O. (2013). Hantavirus infections in Europe and their impact on public health. Rev. Med. Virol. *23*, 35–49.
2. Avsic-Zupanc, T., Petrovec, M., Furlan, P., Kaps, R., Elgh, F., and Lundkvist, A. (1999). Hemorrhagic fever with renal syndrome in the Dolenjska region of Slovenia–a 10-year survey. Clin. Infect. Dis. *28*, 860–865.
3. MacNeil, A., Ksiazek, T.G., and Rollin, P.E. (2011). Hantavirus pulmonary syndrome, United States, 1993-2009. Emerg. Infect. Dis. *17*, 1195–1201.
4. Hjertqvist, M., Klein, S.L., Ahlm, C., and Klingström, J. (2010). Mortality rate patterns for hemorrhagic fever with renal syndrome caused by Puumala virus. Emerg. Infect. Dis. *16*, 1584–1586.
5. Vaheri, A., Strandin, T., Hepojoki, J., Sironen, T., Henttonen, H., Mäkelä, S., and Mustonen, J. (2013). Uncovering the mysteries of hantavirus infections. Nat. Rev. Microbiol. *11*, 539–550.
6. Park, K.H., Kang, Y.U., Kang, S.J., Jung, Y.S., Jang, H.C., and Jung, S.I. (2011). Experience with extrarenal manifestations of hemorrhagic fever with renal syndrome in a tertiary care hospital in South Korea. Am. J. Trop. Med. Hyg. *84*, 229–233.
7. Mustonen, J., Brummer-Korvenkontio, M., Hedman, K., Pasternack, A., Pietilä, K., and Vaheri, A. (1994). Nephropathia epidemica in Finland: a retrospective study of 126 cases. Scand. J. Infect. Dis. *26*, 7–13.
8. Sadeghi, M., Eckerle, I., Daniel, V., Burkhardt, U., Opelz, G., and Schnitzler, P. (2011). Cytokine expression during early and late phase of acute Puumala hantavirus infection. BMC Immunol. *12*, 65.
9. Siamopoulos, K.C., Elisaf, M., Antoniadis, A., and Moutsopoulos, H.M. (1992). Hemorrhagic fever with renal syndrome in an endemic area of Greece. Am. J. Nephrol. *12*, 170–173.
10. Braun, N., Haap, M., Overkamp, D., Kimmel, M., Alscher, M.D., Lehnert, H., and Haas, C.S. (2010). Characterization and outcome following Puumala virus infection: a retrospective analysis of 75 cases. Nephrol. Dial. Transplant. *25*, 2997–3003.
11. Nuutinen, H., Vuoristo, M., Färkkilä, M., Kahri, A., Seppälä, K., Valtonen, V., Joutsiniemi, T., and Miettinen, T. (1992). Hemorrhagic gastropathy in epidemic nephropathy. Gastrointest. Endosc. *38*, 476–480.
12. Klingström, J., Smed-Sörensen, A., Maleki, K.T., Solà-Riera, C., Ahlm, C., Björkström, N.K., and Ljunggren, H.G. (2019). Innate and adaptive immune responses against human Puumala virus infection: immunopathogenesis and suggestions for novel treatment strategies for severe hantavirus-associated syndromes. J. Intern. Med. *285*, 510–523.
13. Krakauer, T., Leduc, J.W., and Krakauer, H. (1995). Serum levels of tumor necrosis factor-alpha, interleukin-1, and interleukin-6 in hemorrhagic fever with renal syndrome. Viral Immunol. *8*, 75–79.
14. Linderholm, M., Ahlm, C., Settergren, B., Waage, A., and Tärnvik, A. (1996). Elevated plasma levels of tumor necrosis factor (TNF)-alpha, soluble TNF receptors, interleukin (IL)-6, and IL-10 in patients with hemorrhagic fever with renal syndrome. J. Infect. Dis. *173*, 38–43.
15. Borges, A.A., Campos, G.M., Moreli, M.L., Moro Souza, R.L., Saggioro, F.P., Figueiredo, G.G., Livonesi, M.C., and Moraes Figueiredo, L.T. (2008). Role of mixed Th1 and Th2 serum cytokines on pathogenesis and prognosis of hantavirus pulmonary syndrome. Microbes Infect. *10*, 1150–1157.
16. Outinen, T.K., Mäkelä, S.M., Ala-Houhala, I.O., Huhtala, H.S., Hurme, M., Paakkala, A.S., Pörsti, I.H., Syrjänen, J.T., and Mustonen, J.T. (2010). The severity of Puumala hantavirus induced nephropathia epidemica can be better evaluated using plasma interleukin-6 than C-reactive protein determinations. BMC Infect. Dis. *10*, 132.
17. Saksida, A., Wraber, B., and Avšič-Županc, T. (2011). Serum levels of inflammatory and regulatory cytokines in patients with hemorrhagic fever with renal syndrome. BMC Infect. Dis. *11*, 142.
18. Kyriakidis, I., and Papa, A. (2013). Serum TNF-α, sTNFR1, IL-6, IL-8 and IL-10 levels in hemorrhagic fever with renal syndrome. Virus Res. *175*, 91–94.

19. Morzunov, S.P., Khaiboullina, S.F., St Jeor, S., Rizvanov, A.A., and Lombardi, V.C. (2015). Multiplex analysis of serum cytokines in humans with hantavirus pulmonary syndrome. Front. Immunol. *6*, 432.

20. Björkström, N.K., Lindgren, T., Stoltz, M., Fauriat, C., Braun, M., Evander, M., Michaëlsson, J., Malmberg, K.J., Klingström, J., Ahlm, C., and Ljunggren, H.G. (2011). Rapid expansion and long-term persistence of elevated NK cell numbers in humans infected with hantavirus. J. Exp. Med. *208*, 13–21.

21. Lindgren, T., Ahlm, C., Mohamed, N., Evander, M., Ljunggren, H.G., and Björkström, N.K. (2011). Longitudinal analysis of the human T cell response during acute hantavirus infection. J. Virol. *85*, 10252–10260.

22. Tuuminen, T., Kekäläinen, E., Mäkelä, S., Ala-Houhala, I., Ennis, F.A., Hedman, K., Mustonen, J., Vaheri, A., and Arstila, T.P. (2007). Human CD8+ T cell memory generation in Puumala hantavirus infection occurs after the acute phase and is associated with boosting of EBV-specific CD8+ memory T cells. J. Immunol. *179*, 1988–1995.

23. Rasmuson, J., Pourazar, J., Linderholm, M., Sandström, T., Blomberg, A., and Ahlm, C. (2011). Presence of activated airway T lymphocytes in human puumala hantavirus disease. Chest *140*, 715–722.

24. Scholz, S., Baharom, F., Rankin, G., Maleki, K.T., Gupta, S., Vangeti, S., Pourazar, J., Discacciati, A., Höijer, J., Bottai, M., et al. (2017). Human hantavirus infection elicits pronounced redistribution of mononuclear phagocytes in peripheral blood and airways. PLoS Pathog. *13*, e1006462.

25. Temonen, M., Mustonen, J., Helin, H., Pasternack, A., Vaheri, A., and Holthöfer, H. (1996). Cytokines, adhesion molecules, and cellular infiltration in nephropathia epidemica kidneys: an immunohistochemical study. Clin. Immunol. Immunopathol. *78*, 47–55.

26. Ma, Y., Wang, J., Yuan, B., Wang, M., Zhang, Y., Xu, Z., Zhang, C., Zhang, Y., Liu, B., Yi, J., et al. (2013). HLA-A2 and B35 restricted hantaan virus nucleoprotein CD8+ T-cell epitope-specific immune response correlates with milder disease in hemorrhagic fever with renal syndrome. PLoS Negl. Trop. Dis. *7*, e2076.

27. Wang, M., Wang, J., Zhu, Y., Xu, Z., Yang, K., Yang, A., and Jin, B. (2009). Cellular immune response to Hantaan virus nucleocapsid protein in the acute phase of hemorrhagic fever with renal syndrome: correlation with disease severity. J. Infect. Dis. *199*, 188–195.

28. Kilpatrick, E.D., Terajima, M., Koster, F.T., Catalina, M.D., Cruz, J., and Ennis, F.A. (2004). Role of specific CD8+ T cells in the severity of a fulminant zoonotic viral hemorrhagic fever, hantavirus pulmonary syndrome. J. Immunol. *172*, 3297–3304.

29. Provine, N.M., and Klenerman, P. (2020). MAIT cells in health and disease. Annu. Rev. Immunol. *38*, 203–228.

30. Corbett, A.J., Eckle, S.B.G., Birkinshaw, R.W., Liu, L., Patel, O., Mahony, J., Chen, Z., Reantragoon, R., Meehan, B., Cao, H., et al. (2014). T-cell activation by transitory neo-antigens derived from distinct microbial pathways. Nature *509*, 361–365.

31. Kjer-Nielsen, L., Patel, O., Corbett, A.J., Le Nours, J., Meehan, B., Liu, L., Bhati, M., Chen, Z., Kostenko, L., Reantragoon, R., et al. (2012). MR1 presents microbial vitamin B metabolites to MAIT cells. Nature *491*, 717–723.

32. Lamichhane, R., Schneider, M., de la Harpe, S.M., Harrop, T.W.R., Hannaway, R.F., Dearden, P.K., Kirman, J.R., Tyndall, J.D.A., Vernall, A.J., and Ussher, J.E. (2019). TCR- or cytokine-activated CD8+ Mucosal-Associated Invariant T Cells are rapid polyfunctional effectors that can coordinate immune responses. Cell Rep. *28*, 3061–3076.e5.

33. Ussher, J.E., Willberg, C.B., and Klenerman, P. (2018). MAIT cells and viruses. Immunol. Cell Biol. *96*, 630–641.

34. van Wilgenburg, B., Scherwitzl, I., Hutchinson, E.C., Leng, T., Kurioka, A., Kulicke, C., de Lara, C., Cole, S., Vasanawathana, S., Limpitikul, W., et al.; STOP-HCV consortium (2016). MAIT cells are activated during human viral infections. Nat. Commun. *7*, 11653.

35. Parrot, T., Gorin, J.B., Ponzetta, A., Maleki, K.T., Kammann, T., Emgård, J., Perez-Potti, A., Sekine, T., and Rivera-Ballesteros, O.; Karolinska COVID-19 Study Group, et al (2020). MAIT cell activation and dynamics associated with COVID-19 disease severity. Sci. Immunol. *5*, eabe1670.

36. Jouan, Y., Guillon, A., Gonzalez, L., Perez, Y., Boisseau, C., Ehrmann, S., Ferreira, M., Daix, T., Jeannet, R., François, B., et al. (2020). Phenotypical and functional alteration of unconventional T cells in severe COVID-19 patients. J. Exp. Med. *217*, e20200872.

37. Lal, K.G., Kim, D., Costanzo, M.C., Creegan, M., Leeansyah, E., Dias, J., Paquin-Proulx, D., Eller, L.A., Schuetz, A., Phuang-Ngern, Y., et al. (2020). Dynamic MAIT cell response with progressively enhanced innateness during acute HIV-1 infection. Nat. Commun. *11*, 272.

38. Flament, H., Rouland, M., Beaudoin, L., Toubal, A., Bertrand, L., Lebourgeois, S., Rousseau, C., Soulard, P., Gouda, Z., Cagninacci, L., et al. (2021). Outcome of SARS-CoV-2 infection is linked to MAIT cell activation and cytotoxicity. Nat. Immunol. Published online February 2, 2021. https://doi.org/10.1038/s41590-021-00870-z.

39. Deschler, S., Kager, J., Erber, J., Fricke, L., Koyumdzhieva, P., Georgieva, A., Lahmer, T., Wiessner, J.R., Voit, F., Schneider, J., et al. (2021). Mucosal-Associated Invariant T (MAIT) cells are highly activated and functionally impaired in COVID-19 patients. Viruses *13*, 241.

40. Hengst, J., Strunz, B., Deterding, K., Ljunggren, H.G., Leeansyah, E., Manns, M.P., Cornberg, M., Sandberg, J.K., Wedemeyer, H., and Björkström, N.K. (2016). Nonreversible MAIT cell-dysfunction in chronic hepatitis C virus infection despite successful interferon-free therapy. Eur. J. Immunol. *46*, 2204–2210.

41. Leeansyah, E., Ganesh, A., Quigley, M.F., Sönnerborg, A., Andersson, J., Hunt, P.W., Somsouk, M., Deeks, S.G., Martin, J.N., Moll, M., et al. (2013). Activation, exhaustion, and persistent decline of the antimicrobial MR1-restricted MAIT-cell population in chronic HIV-1 infection. Blood *121*, 1124–1135.

42. Dias, J., Hengst, J., Parrot, T., Leeansyah, E., Lunemann, S., Malone, D.F.G., Hardtke, S., Strauss, O., Zimmer, C.L., Berglin, L., et al. (2019). Chronic hepatitis delta virus infection leads to functional impairment and severe loss of MAIT cells. J. Hepatol. *71*, 301–312.

43. Fernandez, C.S., Amarasena, T., Kelleher, A.D., Rossjohn, J., McCluskey, J., Godfrey, D.I., and Kent, S.J. (2015). MAIT cells are depleted early but retain functional cytokine expression in HIV infection. Immunol. Cell Biol. *93*, 177–188.

44. van Wilgenburg, B., Loh, L., Chen, Z., Pediongco, T.J., Wang, H., Shi, M., Zhao, Z., Koutsakos, M., Nüssing, S., Sant, S., et al. (2018). MAIT cells contribute to protection against lethal influenza infection in vivo. Nat. Commun. *9*, 4706.

45. Paquin-Proulx, D., Greenspun, B.C., Costa, E.A.S., Segurado, A.C., Kallas, E.G., Nixon, D.F., and Leal, F.E. (2017). MAIT cells are reduced in frequency and functionally impaired in human T lymphotropic virus type 1 infection: Potential clinical implications. PLoS ONE *12*, e0175345.

46. Beudeker, B.J.B., van Oord, G.W., Arends, J.E., Schulze Zur Wiesch, J., van der Heide, M.S., de Knegt, R.J., Verbon, A., Boonstra, A., and Claassen, M.A.A. (2018). Mucosal-associated invariant T-cell frequency and function in blood and liver of HCV mono- and HCV/HIV co-infected patients with advanced fibrosis. Liver Int. *38*, 458–468.

47. Loh, L., Wang, Z., Sant, S., Koutsakos, M., Jegaskanda, S., Corbett, A.J., Liu, L., Fairlie, D.P., Crowe, J., Rossjohn, J., et al. (2016). Human mucosal-associated invariant T cells contribute to antiviral influenza immunity via IL-18-dependent activation. Proc. Natl. Acad. Sci. USA *113*, 10133–10138.

48. Lamichhane, R., Galvin, H., Hannaway, R.F., de la Harpe, S.M., Munro, F., Tyndall, J.D., Vernall, A.J., McCall, J.L., Husain, M., and Ussher, J.E. (2020). Type I interferons are important co-stimulatory signals during T cell receptor mediated human MAIT cell activation. Eur. J. Immunol. *50*, 178–191.

49. Reantragoon, R., Corbett, A.J., Sakala, I.G., Gherardin, N.A., Furness, J.B., Chen, Z., Eckle, S.B., Uldrich, A.P., Birkinshaw, R.W., Patel, O., et al. (2013). Antigen-loaded MR1 tetramers define T cell receptor heterogeneity in mucosal-associated invariant T cells. J. Exp. Med. *210*, 2305–2320.

CellPress
OPEN ACCESS

50. Dias, J., Boulouis, C., Gorin, J.B., van den Biggelaar, R.H.G.A., Lal, K.G., Gibbs, A., Loh, L., Gulam, M.Y., Sia, W.R., Bari, S., et al. (2018). The $CD4^{-}CD8^{-}$ MAIT cell subpopulation is a functionally distinct subset developmentally related to the main $CD8^{+}$ MAIT cell pool. Proc. Natl. Acad. Sci. USA *115*, E11513–E11522.

51. Dias, J., Sobkowiak, M.J., Sandberg, J.K., and Leeansyah, E. (2016). Human MAIT-cell responses to Escherichia coli: activation, cytokine production, proliferation, and cytotoxicity. J. Leukoc. Biol. *100*, 233–240.

52. Freeman, M.L., Morris, S.R., and Lederman, M.M. (2017). CD161 expression on mucosa-associated invariant T cells is reduced in HIV-infected subjects undergoing antiretroviral therapy who do not recover CD4+ T cells. Pathog. Immun. *2*, 335–351.

53. Maleki, K.T., García, M., Iglesias, A., Alonso, D., Ciancaglini, M., Hammar, U., Ljunggren, H.G., Schierloh, P., Martínez, V.P., and Klingström, J. (2019). Serum markers associated with severity and outcome of Hantavirus Pulmonary Syndrome. J. Infect. Dis. *219*, 1832–1840.

54. Outinen, T.K., Laine, O.K., Mäkelä, S., Pörsti, I., Huhtala, H., Vaheri, A., and Mustonen, J. (2016). Thrombocytopenia associates with the severity of inflammation and variables reflecting capillary leakage in Puumala Hantavirus infection, an analysis of 546 Finnish patients. Infect. Dis. (Lond.) *48*, 682–687.

55. Stoltz, M., Ahlm, C., Lundkvist, A., and Klingström, J. (2007). Lambda interferon (IFN-lambda) in serum is decreased in hantavirus-infected patients, and in vitro-established infection is insensitive to treatment with all IFNs and inhibits IFN-gamma-induced nitric oxide production. J. Virol. *81*, 8685–8691.

56. Cosgrove, C., Ussher, J.E., Rauch, A., Gärtner, K., Kurioka, A., Hühn, M.H., Adelmann, K., Kang, Y.H., Fergusson, J.R., Simmonds, P., et al. (2013). Early and nonreversible decrease of CD161++ /MAIT cells in HIV infection. Blood *121*, 951–961.

57. García, M., Iglesias, A., Landoni, V.I., Bellomo, C., Bruno, A., Córdoba, M.T., Balboa, L., Fernández, G.C., Sasiain, M.D., Martínez, V.P., and Schierloh, P. (2017). Massive plasmablast response elicited in the acute phase of hantavirus pulmonary syndrome. Immunology *151*, 122–135.

58. Gupta, S., Braun, M., Tischler, N.D., Stoltz, M., Sundström, K.B., Björkström, N.K., Ljunggren, H.G., and Klingström, J. (2013). Hantavirus-infection confers resistance to cytotoxic lymphocyte-mediated apoptosis. PLoS Pathog. *9*, e1003272.

59. Solà-Riera, C., Gupta, S., Ljunggren, H.G., and Klingström, J. (2019). Orthohantaviruses belonging to three phylogroups all inhibit apoptosis in infected target cells. Sci. Rep. *9*, 834.

60. Chiba, A., Tamura, N., Yoshikiyo, K., Murayama, G., Kitagaichi, M., Yamaji, K., Takasaki, Y., and Miyake, S. (2017). Activation status of mucosal-associated invariant T cells reflects disease activity and pathology of systemic lupus erythematosus. Arthritis Res. Ther. *19*, 58.

61. Emgård, J., Bergsten, H., McCormick, J.K., Barrantes, I., Skrede, S., Sandberg, J.K., and Norrby-Teglund, A. (2019). MAIT cells are major contributors to the cytokine response in group A streptococcal toxic shock syndrome. Proc. Natl. Acad. Sci. USA *116*, 25923–25931.

62. Wensink, A.C., Hack, C.E., and Bovenschen, N. (2015). Granzymes regulate proinflammatory cytokine responses. J. Immunol. *194*, 491–497.

63. Metkar, S.S., Menaa, C., Pardo, J., Wang, B., Wallich, R., Freudenberg, M., Kim, S., Raja, S.M., Shi, L., Simon, M.M., and Froelich, C.J. (2008). Human and mouse granzyme A induce a proinflammatory cytokine response. Immunity *29*, 720–733.

64. Hildebrand, D., Bode, K.A., Rieß, D., Cerny, D., Waldhuber, A., Römmler, F., Strack, J., Korten, S., Orth, J.H., Miethke, T., et al. (2014). Granzyme A produces bioactive IL-1β through a nonapoptotic inflammasome-independent pathway. Cell Rep. *9*, 910–917.

65. Hendel, A., Hsu, I., and Granville, D.J. (2014). Granzyme B releases vascular endothelial growth factor from extracellular matrix and induces vascular permeability. Lab. Invest. *94*, 716–725.

66. Buzza, M.S., Zamurs, L., Sun, J., Bird, C.H., Smith, A.I., Trapani, J.A., Froelich, C.J., Nice, E.C., and Bird, P.I. (2005). Extracellular matrix remodeling by human granzyme B via cleavage of vitronectin, fibronectin, and laminin. J. Biol. Chem. *280*, 23549–23558.

67. Kurioka, A., Jahun, A.S., Hannaway, R.F., Walker, L.J., Fergusson, J.R., Sverremark-Ekström, E., Corbett, A.J., Ussher, J.E., Willberg, C.B., and Klenerman, P. (2017). Shared and distinct phenotypes and functions of human CD161++ Vα7.2+ T cell subsets. Front. Immunol. *8*, 1031.

68. Lazarovits, A.I., Moscicki, R.A., Kurnick, J.T., Camerini, D., Bhan, A.K., Baird, L.G., Erikson, M., and Colvin, R.B. (1984). Lymphocyte activation antigens. I. A monoclonal antibody, anti-Act I, defines a new late lymphocyte activation antigen. J. Immunol. *133*, 1857–1862.

69. Tkachenko, E.A., Bashkirtsev, V.N., van der Groen, G., Dzagurova, T.K., Ivanov, A.P., and Ryltseva, E.V. (1984). Isolation in Vero-E6 cells of Hanta virus from Clethrionomys glareolus captured in the Bashkiria area of the U.S.S.R. Ann. Soc. Belg. Med. Trop. *64*, 425–426.

70. Young, D.F., Andrejeva, L., Livingstone, A., Goodbourn, S., Lamb, R.A., Collins, P.L., Elliott, R.M., and Randall, R.E. (2003). Virus replication in engineered human cells that do not respond to interferons. J. Virol. *77*, 2174–2181.

STAR★METHODS

KEY RESOURCES TABLE

REAGENT or RESOURCE	SOURCE	IDENTIFIER
**Antibodies**		
Mouse monoclonal anti-CD69 BUV395 conjugated	BD Biosciences	Cat#564364, clone FN50
Mouse monoclonal anti-PD-1 BUV737 conjugated	BD Biosciences	Cat#612792, clone EH12.1
Mouse monoclonal anti-Ki67 AF488 conjugated	BioLegend	Cat#350508, clone Ki-67, RRID: AB_10933085
Mouse monoclonal anti-CCR9 AF647 conjugated	BioLegend	Cat#358912, clone L053E8, AB_2562524
Mouse monoclonal anti-granzyme B AF700 conjugated	BD Biosciences	Cat#560213, clone GB11
Mouse monoclonal anti-CD3 APC-Cy7 conjugated	BioLegend	Cat#300426, clone UCHT-1, RRID: AB_830755
Mouse monoclonal anti-CD14 V500 conjugated	BD Biosciences	Cat#561391, clone M5E2
Mouse monoclonal anti-CD19 BV510 conjugated	BD Biosciences	Cat#562947, clone SJ25C1
Mouse monoclonal anti-α4β7 integrin	NIH AIDS Reagent Program, Division of AIDS, NIAID, NIH[68]	Cat#11718
Mouse monoclonal anti-CD8 Qdot605 conjugated	Thermo Fisher Scientific	Cat#Q10009, clone 3B5, RRID: AB_2556437
Mouse monoclonal anti-CD161 BV650 conjugated	BD Biosciences	Cat#563864, clone DX12
Mouse monoclonal anti-CD38 BV711 conjugated	BD Biosciences	Cat#303528, clone HIT2
Mouse monoclonal anti-CCR6 BV786 conjugated	BD Biosciences	Cat#563704, clone 11A9
Mouse monoclonal anti-CD4 PE-Cy5 conjugated	BioLegend	Cat#317412, clone OKT4, RRID: AB_571957
Mouse monoclonal anti-TCR Vα7.2 PE-Cy7 conjugated	BioLegend	Cat#351711, clone 3C10, RRID: AB_2561994
Mouse monoclonal anti-CD107a BUV395 conjugated	BD Biosciences	Cat#565113, clone H4A3
Mouse monoclonal anti-granzyme B FITC conjugated	BioLegend	Cat#515403, clone GB11, RRID: AB_2114575
Mouse monoclonal anti-CD3 AF700 conjugated	BD Biosciences	Cat#557943, clone UCHT1
Mouse monoclonal anti-perforin BV421 conjugated	BioLegend	Cat#353307, clone GB11, RRID:
Mouse monoclonal anti-CD8a BV570 conjugated	BioLegend	Cat#301038, clone RPA-T8, RRID: AB_2563213
Mouse monoclonal anti-CD4 BV711 conjugated	BioLegend	Cat#317440, clone GB11, RRID: AB_2562912
Mouse monoclonal anti-IFN-γ BV785 conjugated	BioLegend	Cat#502542, clone 4S.B3, RRID: AB_2563882
Mouse monoclonal anti-CD69 ECD conjugated	Beckman Coulter	Cat#6607110, clone TP1.55.3
Mouse monoclonal anti-CD161 PE-Cy5 conjugated	BD Biosciences	Cat#551138, clone DX12
Mouse monoclonal anti-IL-17A APC conjugated	BioLegend	Cat#512334, clone BL168, RRID: AB_2563986
Mouse monoclonal anti-TNF BV650 conjugated	BD Biosciences	Cat#563418, clone MAb11
Mouse monoclonal anti-TCR Vα7.2 PE conjugated	BioLegend	Cat#351706, clone 3C10, RRID: AB_10899577
Mouse monoclonal anti-CD25 BUV737 conjugated	BD Biosciences	Cat#612807, clone 2A3
Rat monoclonal anti-GM-CSF APC conjugated	BioLegend	Cat#502309, clone BVD2-21C11, RRID: AB_11148950
Mouse monoclonal anti-IL-12 (p40/p70)	Milteny Biotec	Cat#130-095-755, clone C8.6
Mouse monoclonal anti-IL-18	MBL International Corporation	Cat#DO44-3, clone 125-2H
Mouse monoclonal anti-MR1	BioLegend	Cat#361103, clone 26.5
Mouse monoclonal isotype control IgG1	BioLegend	Cat#401401, clone MG1-45
**Bacterial and virus strains**		
PUUV strain CG-1820	Tkachenko et al.[69]	N/A
**Biological samples**		
PBMC from HFRS patients and controls	Blood bank at the University Hospital of Umeå, Umeå, Sweden	N/A
Plasma from HFRS patients and controls	Blood bank at the University Hospital of Umeå, Umeå, Sweden	N/A

(Continued on next page)

Continued

REAGENT or RESOURCE	SOURCE	IDENTIFIER
Buffy coats from blood donors	Blood Transfusion Clinic at the Karolinska University Hospital Huddinge, Stockholm, Sweden	N/A
Chemicals, peptides, and recombinant proteins		
LIVE/dead^{TM} Fixable aqua dead cell stain kit	Thermo Fisher Scientific	Cat#L34957
Streptavidin QD585 conjugated	Thermo Fisher Scientific	Cat#Q10111MP
MR1 5-OP-RU tetramer BV421 conjugated	NIH Tetramer Core Facility; Corbett et al.[30]	N/A
MR1 5-OP-RU tetramer PE conjugated	NIH Tetramer Core Facility; Corbett et al.[30]	N/A
RosetteSep^{TM} Human monocyte enrichment cocktail	StemCell Technologies, Inc.	Cat#15068
Recombinant human IFN alpha A (alpha 2A)	PBL assay	Cat#11100-1
Recombinant viral B18R protein	R&D Systems	Cat#8185-BR-025
BD GolgiStop^{TM} Protein transport inhibitor (containing monensin)	BD Biosciences	Cat#554724
BD GolgiPlug^{TM} Protein transport inhibitor (containing brefeldin A)	BD Biosciences	Cat#555029
Critical commercial assays		
Human Magnetic Luminex Assay	R&D Systems	Cat#LXSAHM-18, ref: LCeMKDJQ
Human IFN-α pan ELISA development kit (HRP)	Mabtech	Cat#3425-1H-6
Human IL-6 ELISA development kit (HRP)	Mabtech	Cat#3460-1H-6
Human IL-12 ELISA development kit (HRP)	Mabtech	Cat#3455-1H-6
Human TNF ELISA development kit (HRP)	Mabtech	Cat#3511-1H-6
Human IL-15 DuoSet ELISA	R&D Systems	Cat#DY247-05
Human IL-18 DuoSet ELISA	R&D Systems	Cat#DY318-05
Human granzyme B DuoSet ELISA	R&D Systems	Cat#DY2906-05
Human Perforin ELISA Kit	Invitrogen/Thermo Fisher Scientific	Cat#BMS2306
eBioscience Foxp3/Transcription Factor Staining Buffer Set	eBioscience	Cat#00-5523-00
Transcription Factor Buffer Set	BD Biosciences	Cat#562725
FluoReporter Mini-biotin-XX Protein Labeling Kit	Invitrogen/Thermo Fisher Scientific	Cat#F6347
Experimental models: cell lines		
Human: A549-V cells	[70]	N/A
African green monkey: Vero E6	ATCC	Cat#CRL-1586, RRID: CVCL_0574
Human: HUVEC	Lonza	Cat#CC-2519, batch: 0000474578
Human: THP-1	ATCC	Cat#TIB-202, RRID: CVCL_0006
Software and algorithms		
FlowJo version 10.7.1	Tree Star Inc.	https://www.flowjo.com
GraphPad Prism 9.0.0	GraphPad Software	https://www.graphpad.com

RESOURCE AVAILABILITY

Lead contact

Further information and requests for resources and reagents may be directed to the Lead Contact, Kimia T. Maleki (kimia.maleki@ki.se).

Materials availability

This study did not generate new unique reagents.

Data and code availability

This study did not generate or analyze datasets or code.

EXPERIMENTAL MODEL AND SUBJECT DETAILS

Patient samples

Twenty-four Swedish HFRS patients and 19 community-matched uninfected controls were included in the study. The patients were diagnosed with PUUV infection at Umeå University Hospital in Umeå, Sweden, during the years 2006-2014. PBMCs were isolated from whole blood using CPT tubes, as previously described,[24] and stored in liquid nitrogen until analysis. Plasma was collected and stored at -80°C until analysis. Written consent was obtained from all subjects before participation. Ethical approval for the study was obtained from the Regional Ethics Committee of Umeå University (application number 04-133M).

Patient characteristics and clinical data are summarized in Table 1. The 24 HFRS patients included 9 females and 15 males of a mean age of 49 years (range 29-67 years) and the controls included 6 females and 13 males of a mean age of 50 years (range 38-70 years). Samples from the acute, intermediate, and convalescent phase were collected from HFRS patients at a median of 6 days (range 3-9 days), 13 days (range 10-21 days), and 130 days (range 42-574 days) post symptom debut, respectively. Intermediate samples were available for 21 of the 24 HFRS patients.

Cells and viruses

PBMCs for *in vitro* studies were isolated from buffy coats obtained from Karolinska University Hospital (Stockholm, Sweden) using Lymphoprep (StemCell Technologies, Inc.). Cells were rested overnight in RPMI-1640 medium (GE Healthcare) supplemented with 25 mM HEPES (GE Healthcare), 2 mM L-glutamine (Thermo Fisher Scientific), 10% FCS (Sigma-Aldrich), 50 μg/mL gentamicin (Life Technologies), and 100 μg/mL normocin (InvivoGen). TCR Vα7.2$^+$ cells were isolated from PBMCs by positive selection using PE-conjugated anti-TCR Vα7.2 antibody and anti-PE MACS microbeads (Milteny Biotec), according to the manufacturer's instructions. Monocytes were enriched from PBMCs isolated from blood donors using RosetteSep Monocyte Enrichment Cocktail (StemCell Technologies, Inc.). Primary monocytes and THP-1 cells (sex: male) (ATCC) were maintained in RPMI-1640 medium supplemented as described above. Primary human umbilical vein endothelial cells (HUVECs) (pooled donors, sex: mixed) (Lonza) were maintained in EGM-2 medium supplemented according to the manufacturer's guidelines (Lonza), with the exception that cortisone was only included until cells were split to plates used for infection experiments. Human A549-V cells[70] (sex: male) and African green monkey VeroE6 cells (sex: female) were grown in MEM supplemented with 5% FCS, 2 mM L-glutamine, and penicillin-streptomycin (Thermo Fisher Scientific). All cells were cultured at 37°C in 5% CO_2.

PUUV strain CG-1820[69] was propagated on A549-V[70] cells as previously described for Vero E6 cells.[55] Supernatants were pelleted by ultracentrifugation and then viruses were resuspended in MEM supplemented as described above, aliquoted and stored at -80°C. Viral titer was determined by titration on Vero E6 cells, as previously described.[55]

METHOD DETAILS

In vitro PUUV stimulation of PBMCs

PBMCs (1x10^6 cells per well in a 96 well plate) were exposed to PUUV at a multiplicity of infection (MOI) 1 for 2 h, or left unstimulated, at 37°C and then washed once. Anti-CD107a antibody was added to the cells at the start of the cultures. After 72 h incubation at 37°C, cells were centrifuged and stained for flow cytometric analyses.

In vitro stimulation of MAIT cells using THP-1 cells

THP-1 cells (0.2x10^6 cells per well in a 96 well plate) were exposed to PUUV at MOI 5 for 3 h, or left unstimulated, at 37°C and then washed once. PUUV was UV-inactivated using a VL215G Vilber Lourmat UV lamp (Torcy) for 25 s and used as a control. After 72 h, isolated TCR Vα7.2$^+$ cells (0.2x10^6 cells) were added to the THP-1 cells. Blocking antibodies (5 μg/ml) were added to the THP-1 cells 2 h before start of the co-culture, while B18R (1 μg/ml) was added 10-12 h before start of the co-cultures. Anti-CD107a antibody was added at the start of co-cultures. After 12 h of co-culture, supernatants were collected and cells were stained for flow cytometry.

In vitro stimulation of MAIT cells using primary cell supernatants

Primary monocytes (0.2x10^6 cells per well in a 96 well plate) were exposed to PUUV at MOI 1 for 2 h. HUVECs (0.2x10^6 cells per well in a 24 well plate) were infected with PUUV for 1 h, with gentle shaking every 15 min. Monocytes and HUVECs were then washed and resuspended in fresh medium. After 48 h, cell culture supernatants from unstimulated and PUUV-exposed monocytes and HUVECs were collected and either used directly or frozen at -80°C. B18R was added to the collected supernatants 4-5 h prior to experiments. TCR Vα7.2$^+$ cells (0.2x10^6 cells per well in a 96 well plate) were resuspended in conditioned medium from monocytes or HUVECs, with or without B18R, and maintained at 37°C. After 24 h, supernatants were collected, and cells were stained for flow cytometry. TCR Vα7.2$^+$ cells stimulated with recombinant IFN-α_2 (1000 U/ml) (PBL assay) were used as control.

Biotinylation assay

α4β7 integrin antibodies were biotinylated using FlouReporter Mini-biotin-XX Protein Labeling Kit (Invitrogen), according to the manufacturer's protocol.

Flow cytometric analyses

Brefeldin A (Golgi Plug, BD Biosciences) and monensin (Golgi Stop, BD Biosciences) were added to cultures 4-5 h prior to staining. The following antibodies/conjugates were used for phenotypical characterization of MAIT cells in HFRS patients and controls: CD69-BUV395, PD-1-BUV737, granzyme B-AF700, CD14-V500, CD19-BV510, CD161-BV650, CCR6-BV786, (all from BD Biosciences), Ki67-AF488, CCR9-AF647, CD3-APC-Cy7, CD38-BV711, CD4-PE-Cy5, TCR Vα7.2-PE-Cy7 (all from BioLegend), α4β7 integrin (NIH),[68] streptavidin-QD585, and CD8-QD605 (Thermo Fisher Scientific). PBMCs from patients and controls were washed once after thawing, and then stained with MR1 5-OP-RU tetramer-BV421[30] for 40 min at room temperature. Extracellular staining was performed for 30 min at room temperature. Dead cells were identified using Live/Dead Aqua (Invitrogen). Cells were fixed and permeabilized using Transcription Factor Staining Buffer Set (eBioscience) and intracellular staining was performed for 60 min at room temperature.

The following antibodies were used for characterization of MAIT cells in *in vitro* experiments: CD107a-BUV395, CD25-BUV737, CD3-AF700, CD161-PE-Cy5, TNF-BV650 (all from BD Biosciences), granzyme B-FITC, perforin-BV421, CD8-BV570, CD38-BV650, CD4-BV711, IFN-γ-BV785, TCR Vα7.2-PE-Cy7, TCR Vα7.2-PE, IL-17A-APC, GM-CSF-APC (all from BioLegend), and CD69-ECD (Beckman Coulter). Cells were stained with MR1-OP-RU tetramer-PE[30] for 40 min at room temperature, followed by extracellular staining with antibodies for 20 min at 4°C. Cells were fixed and permeabilized using Transcription factor buffer set (BD Biosciences) for 30 min at 4°C before intracellular staining for 30 min at 4°C. For experiments with purified TCR Vα7.2$^+$ cells, no tetramer was used.

All samples were acquired on a BD LSR Fortessa instrument (BD Biosciences) equipped with 355, 405, 488, 561, and 639 nm lasers. Data were analyzed using FlowJo version 10.7.1. (Tree Star Inc.).

Multiplex immunoassay

Plasma levels of IL-1β, IL-2, IL-6, IL-10, IL-12 (p70), IL-15, IL-17A, IL-17C, IL-17E, IL-18, IL-22, TNF, IFN-γ, CCL20, CCL25, granzyme A, and granzyme B were measured in plasma diluted 1:2, using a custom Magnetic Luminex Screening assay (R&D Systems).

ELISA

IFN-α levels in cell culture supernatants and patient plasma were analyzed using human IFN-α pan ELISA development kit (Mabtech) according to the manufacturer's guidelines. Plasma was diluted 1:3 in ready-to-use ELISA diluent (Mabtech) prior to IFN-α ELISA. Levels of IL-6, TNF, and IL-12 in supernatants were analyzed using ELISA development kits (Mabtech) and levels of IL-15, IL-18, and granzyme B in supernatants were analyzed using DuoSet ELISA kits (R&D). Perforin levels were assessed using human perforin ELISA kit (Invitrogen), according to the manufacturer's instructions.

QUANTIFICATION AND STATISTICAL ANALYSIS

Statistical analyses were performed using Graph Pad Prism v.9. Kruskal-Wallis test with Dunn's multiple comparison test was used for statistical comparisons between controls and HFRS patients. Paired comparisons within HFRS patients or between more than two *in vitro* conditions were performed using Friedman's test with Dunn's multiple comparison test. Wilcoxon signed-rank test was used for pairwise comparisons of two groups. Spearman's rank correlation coefficient was used for examining correlations. Bar graphs show median. n-values are indicated in the figure legends and refer to number of patients or blood donors.

Cell Reports Medicine, Volume *2*

Supplemental information

MAIT cell activation is associated with disease severity markers in acute hantavirus infection

Kimia T. Maleki, Johanna Tauriainen, Marina García, Priscilla F. Kerkman, Wanda Christ, Joana Dias, Julia Wigren Byström, Edwin Leeansyah, Mattias N. Forsell, Hans-Gustaf Ljunggren, Clas Ahlm, Niklas K. Björkström, Johan K. Sandberg, and Jonas Klingström

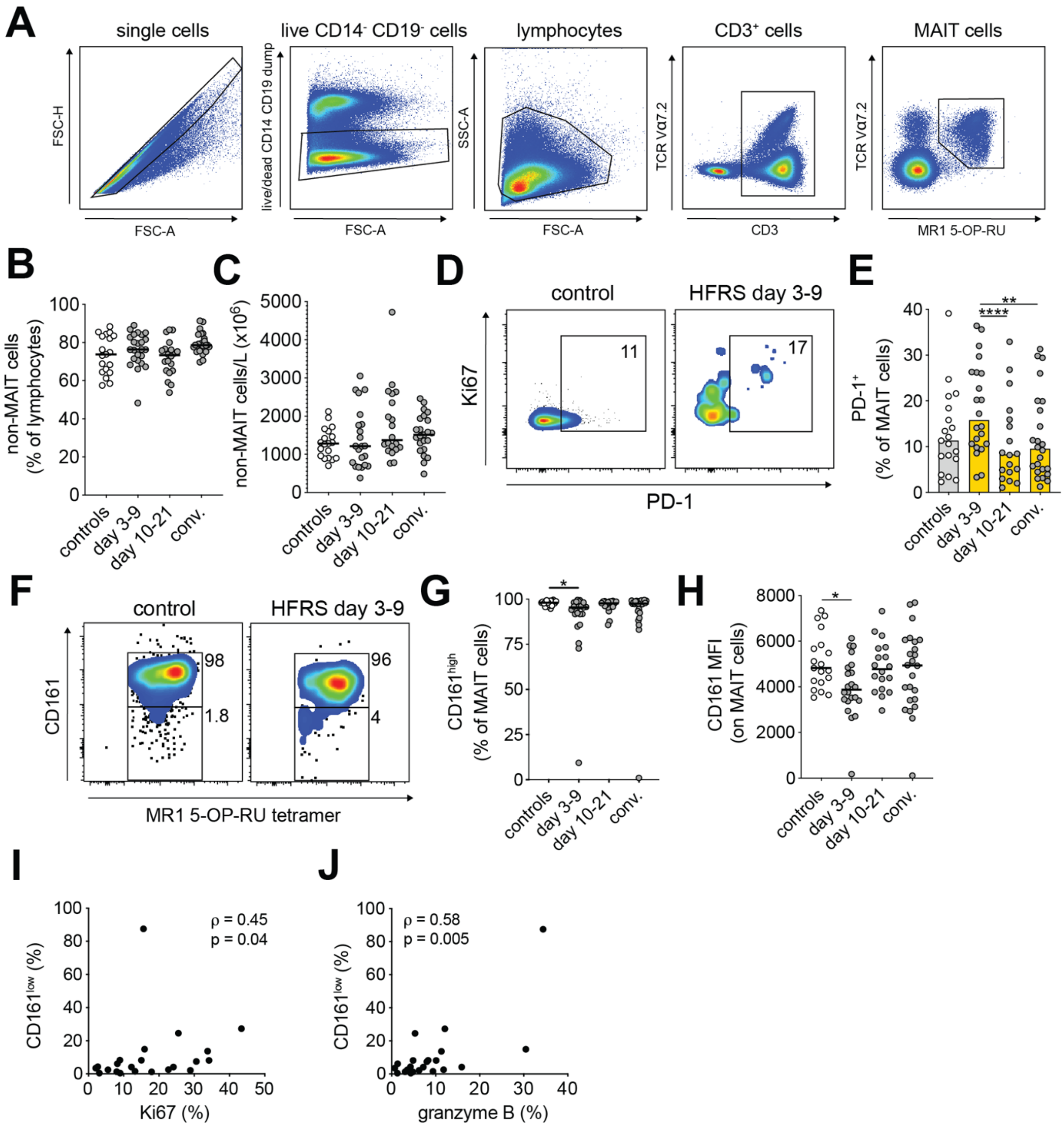

Figure S1. Gating strategy, non-MAIT T cell levels and MAIT cell surface marker expression in blood of HFRS patients and controls. Related to Figures 1-4. (A) Gating strategy for identification of MR1 5-OP-RU tetramer$^+$ MAIT cells in PBMCs. (B) Frequency and (C) absolute count of non-MAIT T cells in controls and HFRS patients during acute, intermediate and convalescent phase. (D) Representative flow cytometry plots showing frequencies of PD-1$^+$ MAIT cells in a control and in a patient with acute HFRS. (E) Frequencies of PD-1$^+$ MAIT cells in controls and HFRS patients. (F) Representative flow cytometry plots of the frequencies of CD161^{high} and CD161^{low} MAIT cells in a control and a patient with acute HFRS. (G) Frequencies of CD161^{high} MAIT cells and (H) MFI of CD161 on MAIT cells in controls and HFRS patients. (I) Correlation between the frequency of CD161^{low} MAIT cells and the expression of Ki67 and (J) granzyme B on MAIT cells during acute HFRS. Acute phase, day 3-9 (n=22-24); intermediate phase, day 10-21 (n=18-21); convalescent phase (conv., n=24). Horizontal lines and bars represent median values. Kruskal Wallis test, Friedman test. *p < 0.05, **p < 0.01, ****p <0.0001. Spearman's rank correlation coefficient.

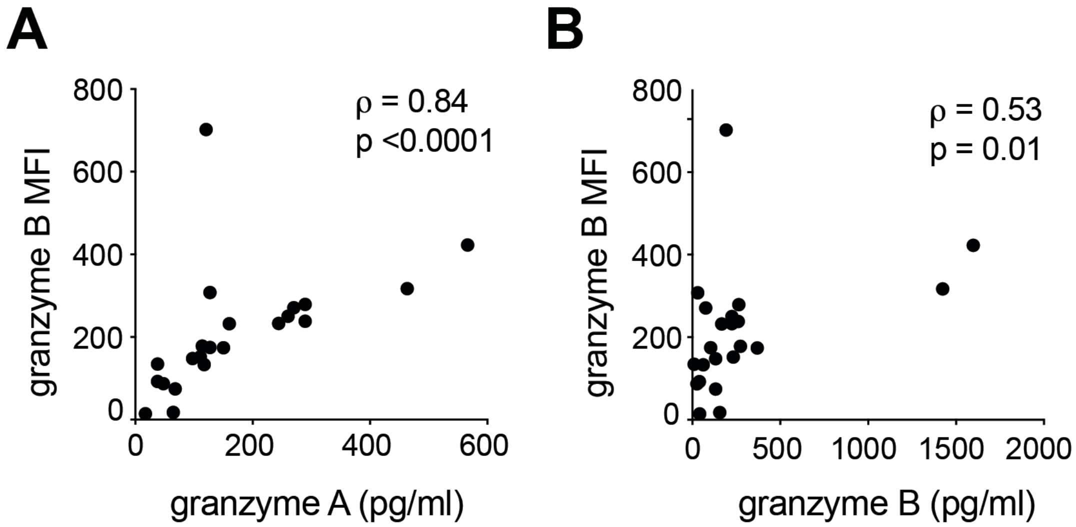

Figure S2. MAIT cell granzyme B expression correlates with levels of plasma granzymes. Related to Figures 2-3. n=22. Spearman's rank correlation coefficient.

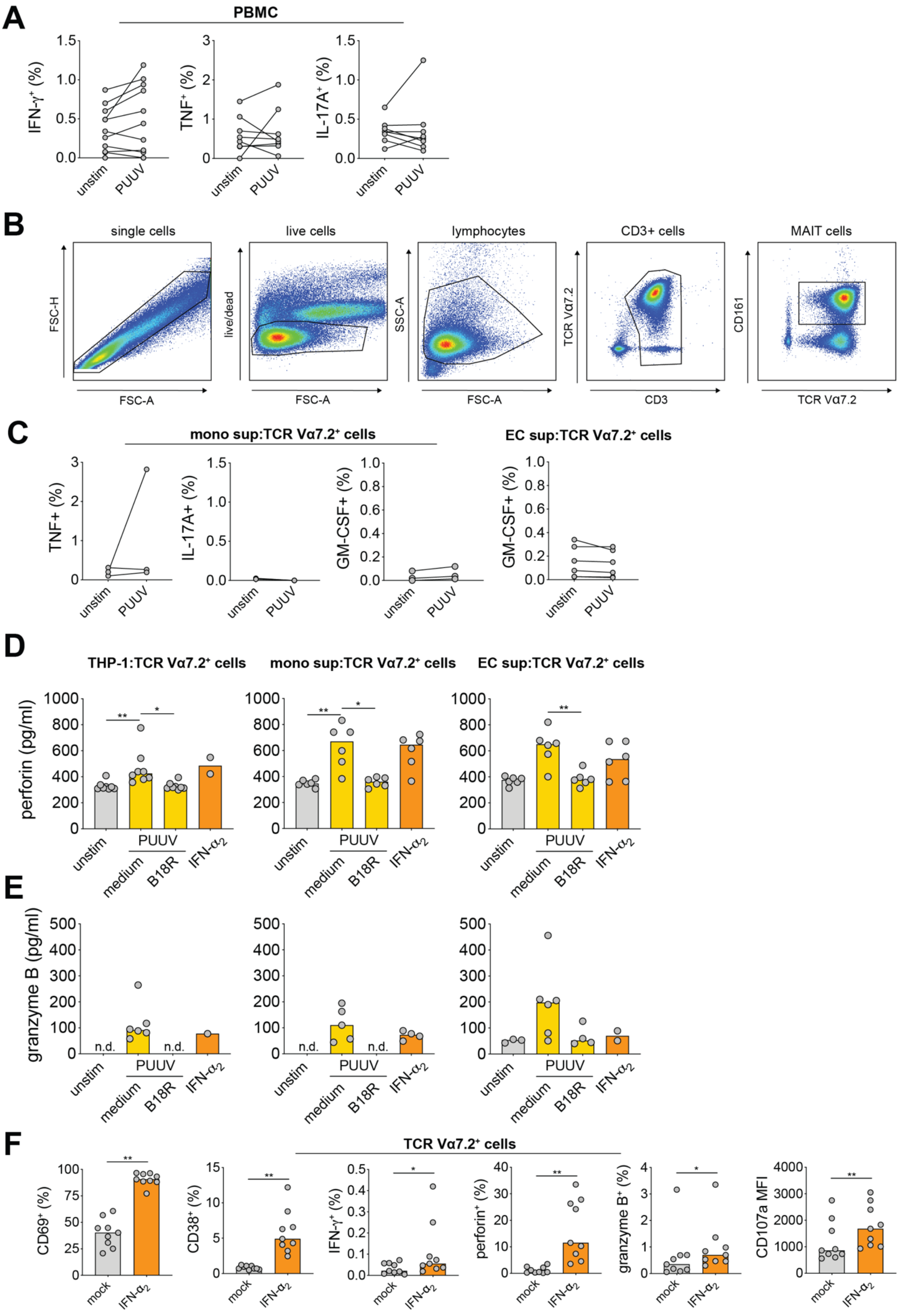

Figure S3. Gating strategy, cytokine expression and release of cytotoxic granule content by MAIT cells in *in vitro* studies. Related to Figures 5-6. (A) IFN-γ, TNF, and IL-17A expression on MAIT cells within PBMCs (n=8-11, at least three independent experiments) with or without PUUV for 72 h. (B) Gating strategy for

identification of MAIT cells among purified TCR Vα7.2$^+$ cells. (C) TNF, IL-17A, and/or GM-CSF expression on MAIT cells after culture with conditioned medium from primary monocytes (mono) (n=3, one experiment) or endothelial cells (EC) (n=6, two independent experiments). (D) Levels of perforin and (E) granzyme B released into supernatants from TCR Vα7.2$^+$ cells cultured with THP-1 cells (n=8, three independent experiments), supernatants from primary monocytes (n=6, two independent experiments) or endothelial cells (n=6, two independent experiments), or together with recombinant IFN-α_2 (1000 U/ml). Samples with undetectable granzyme B concentrations are not shown in the figure. (F) Expression of CD69, CD38, IFN-γ, perforin, granzyme B, and CD107a on MAIT cells (n=9, three independent experiments) with or without recombinant IFN-α_2 stimulation for 24 h. Bars represent median values. Wilcoxon test, Friedman test. *$p < 0.05$, **$p < 0.01$. n.d., not detected.
